# Examining the variability of neurocognitive functioning in individuals at clinical high risk for psychosis: a meta-analysis

**DOI:** 10.1038/s41398-022-01961-7

**Published:** 2022-05-12

**Authors:** Ana Catalan, Joaquim Radua, Robert McCutcheon, Claudia Aymerich, Borja Pedruzo, Miguel Ángel González-Torres, Helen Baldwin, William S. Stone, Anthony J. Giuliano, Philip McGuire, Paolo Fusar-Poli

**Affiliations:** 1 Mental Health Department. Basurto University Hospital. Biocruces Bizkaia Health Research Institute. Department of Neuroscience, Campus de Leioa, University of the Basque Country, UPV/EHU. Plaza de Cruces 12. 48903, Barakaldo, Bizkaia Spain; 2 Early Psychosis: Interventions and Clinical-detection (EPIC) Lab, Department of Psychosis Studies, Institute of Psychiatry, Psychology & Neuroscience, King’s College London, London, UK; 3 Imaging of Mood- and Anxiety-Related Disorders (IMARD) Group, Mental Health Research Networking Center (CIBERSAM), Institut d’Investigacions Biomèdiques August Pi i Sunyer (IDIBAPS), Barcelona, Spain; 4 Department of Clinical Neuroscience, Centre for Psychiatric Research and Education, Karolinska Institutet, Stockholm, Sweden; 5 Department of Psychosis Studies, King’s College London, London, UK; 6 Psychiatry Department, Basurto University Hospital, Bilbao, Spain; 7 Department of Psychiatry, Beth Israel Deaconess Medical Center, Harvard Medical School, Boston, MA USA; 8 Worcester Recovery Center & Hospital, Massachusetts Department of Mental Health, Boston, MA USA; 9 Department of Psychosis Studies, Institute of Psychiatry, Psychology and Neuroscience, King’s College London, London, UK; 10 Department of Brain and Behavioral Sciences, University of Pavia, Pavia, Italy; 11 National Institute for Health Research (NIHR) Biomedical Research Centre (BRC), London, UK; 12 Outreach and Support in South London (OASIS) service, South London and Maudsley NHS Foundation Trust, London, UK

**Keywords:** Diagnostic markers, Schizophrenia

## Abstract

This study aims to meta-analytically characterize the presence and magnitude of within-group variability across neurocognitive functioning in young people at Clinical High-Risk for psychosis (CHR-P) and comparison groups. Multistep, PRISMA/MOOSE-compliant systematic review (PROSPERO-CRD42020192826) of the Web of Science database, Cochrane Central Register of Reviews and Ovid/PsycINFO and trial registries up to July 1, 2020. The risk of bias was assessed using a modified version of the NOS for cohort and cross-sectional studies. Original studies reporting neurocognitive functioning in individuals at CHR-P compared to healthy controls (HC) or first-episode psychosis (FEP) patients were included. The primary outcome was the random-effect meta-analytic variability ratios (VR). Secondary outcomes included the coefficient of variation ratios (CVR). Seventy-eight studies were included, relating to 5162 CHR-P individuals, 2865 HC and 486 FEP. The CHR-P group demonstrated higher variability compared to HC (in descending order of magnitude) in visual memory (VR: 1.41, 95% CI 1.02–1.94), executive functioning (VR: 1.31, 95% CI 1.18–1.45), verbal learning (VR: 1.29, 95% CI 1.15–1.45), premorbid IQ (VR: 1.27, 95% CI 1.09–1.49), processing speed (VR: 1.26, 95% CI 1.07–1.48), visual learning (VR: 1.20, 95% CI 1.07–1.34), and reasoning and problem solving (VR: 1.17, 95% CI 1.03–1.34). In the CVR analyses the variability in CHR-P population remains in the previous neurocognitive domains and emerged in attention/vigilance, working memory, social cognition, and visuospatial ability. The CHR-P group transitioning to psychosis showed greater VR in executive functioning compared to those not developing psychosis and compared to FEP groups. Clinical high risk for psychosis subjects shows increased variability in neurocognitive performance compared to HC. The main limitation of this study is the validity of the VR and CVR as an index of variability which has received debate. This finding should be explored by further individual-participant data research and support precision medicine approaches.

## Introduction

Compromised neurocognition is well-established as a core feature of psychotic disorders and is reliable though heterogeneously present in all phases of the illness [1–6], including premorbid stages [7] and clinical high-risk states for psychosis (CHR-P) [8]. CHR-P individuals have 20% likelihood of developing psychosis at 2-years which is not affected by the age at presentation or psychometric instruments used [9–11]. CHR-P individuals as a group tend to demonstrate a neurocognitive deficit severity that is intermediary to healthy controls (HC) and those with the first episode of psychosis (FEP) [8, 12], and some neurocognitive deficits are reliably associated with the longitudinal transition to psychosis [8]. Moreover, neurocognitive impairments associated with psychotic disorders and their risk states are among the most significant and consistent predictors of functional outcomes in both cross-sectional and longitudinal studies [13–16].

At the same time, it is equally well known that premorbid-developmental stages and diagnostic-clinical and functional outcomes among psychosis-spectrum groups at all stages are notably heterogeneous [17]. The challenge of variability, and its impact on treatment responsiveness and prediction of longitudinal trajectories, is common across psychiatry research, and it has only recently been re-engaged as a focus of quantitative study. Inter-individual neurocognitive variability within psychosis-spectrum groups has been characterized by data-driven cluster analyses and commonly reveals three to four subgroups among adults with established illness [18, 19]. In general, subgroups of individuals with schizophrenia-spectrum and bipolar disorders are most distinguished by differential levels of neurocognitive functioning (i.e. a near-normal, “spared” or intact cognition group, a globally or severely impaired group, and one or two subgroups with graded deficits across neurocognitive domains and often referred to as “selectively” impaired or “intermediate” subtypes falling in between the extremes of near-normal and severely impaired [20–23]. Substantial heterogeneity has been previously demonstrated for the CHR-P paradigm. A meta-analysis from our group showed high variability in the level of likelihood of transitioning to psychosis across CHR-P individuals, with those presenting with a Brief and Limited Intermittent Psychotic Episode having a substantially higher risk than other subgroups [24–28]. There is further evidence showing that most of CHR-P clinical heterogeneity is accounted for by the recruitment and sampling phase by selecting individuals undergoing a CHR-P assessment [29–31]. The challenge of clinical heterogeneity in CHR-P groups has become a mainstream focus of clinical research promoting the development of precision medicine approaches and the initiation of large-scale international research programmes to deconstruct it [32–34].

We recently completed a meta-analysis quantifying group mean differences in levels of performance across neurocognitive domains and tests. This study demonstrated a worse group-level neurocognitive performance in the olfaction, verbal learning, reasoning and problem solving, visual memory, verbal memory, working memory, visual learning, executive functioning, general intelligence, processing speed, attention or vigilance, premorbid intelligence, visuospatial ability, social cognition, and motor functioning domains for CHR-P individuals compared to HC [8]. And a better performance in the general intelligence, verbal learning and executive functioning neurocognitive domains compared to first-episode psychosis (FEP). This meta-analyiss also found that those CHR-P individuals developing psychosis had worse neurocognitive functioning in the verbal learning, visual memory, processing speed, attention or vigilance, and general intelligence domains compared to those not developing psychosis.

Overall, these findings extend previous neurocognitive meta-analyses in this area [8, 35]. Yet, these studies did not test whether these estimates are meaningfully representing a largely universal average effect across this heterogeneous population or whether it is confined to a subgroup of CHR-P individuals. [8, 35, 36].

The current study aims to expand our prior meta-analysis by studying the degree of inter-individual variability within CHR-P groups compared to HC and FEP groups. In other words, we hypothesize that the CHR-P groups may not only differ from HC and FEP in terms of their means but also their variances. Meta-analyses of variability has been used previously to study the immune system [37], brain volumes [38, 39], treatment response [40–43], and clinical features [44] in patients and provide additional evidence of subgroups. Furthermore, we have previously investigated variability in the response to preventive treatments in CHR-P populations [45]. More specifically, heterogeneity/homogeneity of a certain estimate can be quantified using the variability ratio (VR) and the coefficient of variation (CVR) [46, 47] in patient groups compared to controls. To our knowledge, these methods have never been applied to examine the variability of neurocognitive functioning in the CHR-P population. Given the high clinical variability observed in the CHR-P group we hypothesized a higher neurocognitive variability in CHR-P individuals compared to HC.

## Methods

This review (study protocol registered on PROSPERO-CRD42020192826) was conducted following the Preferred Reporting Items for Systematic Reviews and Meta-Analyses (PRISMA, eTable 1) [48], MOOSE (eTable 2) [49], and EQUATOR guidelines [50]. The results of the initial meta-analytic review, in terms of Hedges´g, have been previously described [8]. The search terms are detailed in eMethods 1.

### Outcome measures and data extraction

A detailed description of the extraction procedure and neurocognitive tests used are explained elsewhere [8] (eTable 3). Neurocognitive tasks were organized in accord with the 7 MATRICS Consensus Cognitive Battery and 8 additional domains or tests within domains as detailed in eTable 3. To merge the non-independent neurocognitive tasks, we used a previously described method [8]. Primary outcome measures included the VR in the whole CHR-P group, CHR-P developing psychosis or not, controls and FEP. Secondary outcome measures included the CVR calculations in the whole CHR-P group, CHR-P developing psychosis or not, HC and FEP.

### Statistical analyses

The primary analysis investigated the VR (the logarithm of the ratio of the standard deviations (SDs), named log “variability ratio”) across the different neurocognitive domains in CHR-P individuals compared to HC, between CHR-P developing psychosis and not developing psychosis and between CHR-P and first-episode psychosis (FEP). Given the presence of between-group mean variability in neurocognitive performance, we also conducted secondary mean-scaled CVR analyses (the logarithm of the ratio of the coefficients of variation (CV), termed the log “CV ratio”) to validate the VR analyses [51], again comparing CHR-P individuals vs HC, CHR-P transitioning vs those not transitioning and CHR-P individuals vs FEP. Comparative tests and effect sizes were reported for each neurocognitive task and then across each larger neurocognitive domain. Overall, our analyses yielded an in-depth characterization of neurocognitive variability in this dataset using baseline clinical and longitudinal clinical outcomes (i.e. the transition to psychosis).

The VR has gained recent attention as an indicator of inter-individual variability for various clinical factors, such as treatment effect [45, 46] and its logarithm (needed to approach normality) was calculated according to the formula below:

$$\begin{array}{l}{\it{ln}}\quad VR = {{{\mathrm{log}}}}\left( {\frac{{SD_{Tx}}}{{SD_{Ct}}}} \right) + \frac{1}{{2 \cdot \left( {n_{Tx} - 1} \right)}} - \frac{1}{{2 \cdot \left( {n_{Ct} - 1} \right)}}\\ SE_{{\mathrm{log}}VR} = \sqrt {\frac{1}{{2 \cdot \left( {n_{Tx} - 1} \right)}} + \frac{1}{{2 \cdot \left( {n_{Ct} - 1} \right)}}} \end{array}$$

n_Tx_ and SD_Tx_ are the sample size and standard deviation in the CHR-P (and transition) group, while n_Ct_ and SD_Ct_ are the sample size and standard deviation in the comparative groups (e.g. HC, FEP, or CHR-P not developing psychosis).

If different subgroups of CHR-P individuals with different neurocognitive profiles do not exist, the variability in this group should be similar to that observed in comparative groups (e.g. HC). Therefore, a VR of 1 demonstrates equal variability in neurocognitive tasks between those at CHR-P and HC (or other comparative groups). A VR greater than 1 suggests greater variability in the neurocognitive tasks of those in the CHR-P group (or other comparative groups), whereas a VR less than 1 indicates less variability in the CHR-P group (or other comparative groups). The log-VR was back-transformed into the linear scale (VR) to aid the interpretation of the results. For all calculations, we set an a priori significance threshold of *p* < 0.05. Lastly, we conducted a standard random-effects meta-analysis with restricted maximum likelihood estimation of the heterogeneity to pool the different studies’ logCV (Coefficient of Variation for Log-transformed Data). We exponentiated the log-VR in the forest plot to back-transform from the log scale for the benefit of interpretability.

Metaregressions evaluated the impact of several factors: age, sex, years of education, ethnicity, global functioning (measured by Global Assessment Functioning scale), attenuated positive and negative psychotic symptoms (measured by the SIPS, Structured Interview for Psychosis-Risk Syndromes), NOS (Newcastle Ottawa Scale) quality scale [11, 31], baseline antipsychotic exposure and type of CHR-P instrument used). Metaregressions were conducted when at least 7 studies were available.

All analyses were conducted within R 1.4.1106 [52], the VR and CVR analyses were performed using the metafor package [53].

## Results

### Characteristics of the dataset

**Fig. 1:**
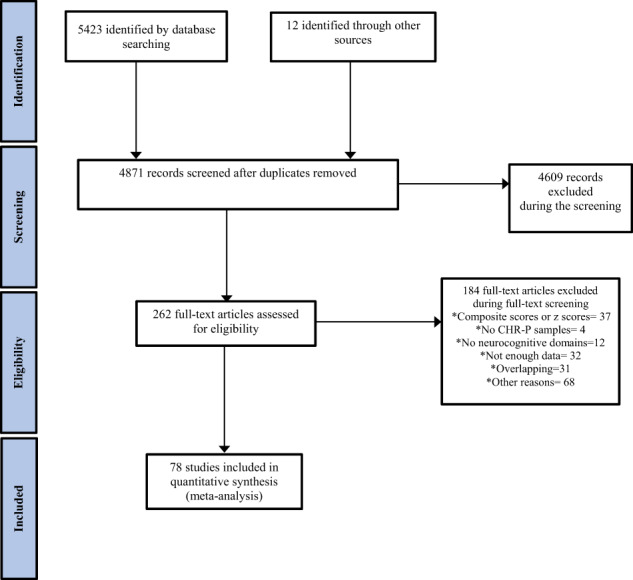
PRISMA Flow Diagram. Preferred Reporting Items for Systematic Reviews and Meta-Analyses (PRISMA) flowchart outlining the study selection process.

A total of 262 eligible studies were screened; 78 of them were included (Fig. 1 and eTable 5) comprising 5162 CHR-P individuals (mean age 20.16 years, SD = 3.25, range 12–29.01, 49% females), 2865 HC (mean age 21.07 years, SD = 3.56, range 12.58–29.23, 52% females) and 486 FEP individuals (mean age 23.03, SD = 2.01, range 19.1–26.4, 55% females). Within the CHR-P group, 71.81% fulfilled attenuated psychotic symptoms (APS) criteria, 7.24% brief limited intermittent psychotic symptoms (BLIPS) criteria, 13.57% genetic risk and deterioration syndrome (GRD) and 7.39% basic symptoms (BS). At baseline, 19.9% of CHR-P individuals had been treated with antipsychotic medication (at any dosage).

### VR and CVR variability of neurocognitive functioning: CHR-P vs HC

The pooled VR neurocognitive domains are shown in Table 1. The CHR-P group presented greater variability than HC across most neurocognitive domains, including (in decreasing order of magnitude) visual memory (VR: 1.41, 95% CI 1.02, 1.94), executive functioning (VR: 1.31, 95% CI 1.18, 1.45), verbal learning (VR: 1.29, 95% CI 1.15, 1.45), premorbid IQ (VR: 1.27, 95% 1.09, 1.49), processing speed (VR: 1.26, 95% CI 1.07, 1.48), visual learning (VR: 1.20, 95% CI 1.07, 1.34), and reasoning and problem solving (VR: 1.17, 95% CI 1.03, 1.34). In the CVR analyses, these results remained similar with greater variability for the CHR-P group in visual memory (CVR: 2.27, 95% CI 1.00, 5.13), verbal learning (CVR: 1.45, 95% CI 1.29, 1.62), processing speed (CVR: 1.42, 95% CI 1.21, 1.67), premorbid IQ (CVR: 1.33, 95% CI 1.11, 1.58), visual learning (CVR: 1.30, 95% CI 1.14, 1.48), social cognition (CVR: 1.19, 95% CI 1.03, 1.37), attention/vigilance (CVR: 1.17, 95% CI 1.08, 1.26), reasoning and problem solving (CVR: 1.17, 95% CI 1.03, 1.34), visuospatial ability (CVR: 1.17, 95% CI 1.08, 1.27), executive functioning (CVR: 1.15, 95% CI 1.01, 1.31), and working memory (CVR: 1.14, 95% CI 1.07, 1.21) (Table 1).

**Table 1:** VR and CVR in different neurocognitive domains between CHR-P vs HC, CHR-P transitioned vs CHR-P non-transitioned, and CHR-P vs FEP; CHR-P Clinical High Risk for Psychosis; HC Healthy Controls; FEP First Episode Psychosis.

Neurocognitive domains	VR	95% CI []	SE	Z	*p*	I^2^	Q	CVR	95% CI []	SE	Z	*p*
**CHR-P vs HC**												
Processing speed	1.26	[1.07, 1.48]	0.0828	2.7722	**0.0056**	90.8%	180.9011, *p* < 0.0001	1.42	[1.21, 1.67]	0.028	4.2388	**<0.0001**
Attention/vigilance	1.01	[0.94, 1.08]	0.0347	0.1614	0.871	99.25%	1616.6610, *p* < 0.0001	1.17	[1.08, 1.26]	0.0375	41.069	**<0.0001**
Working memory	1.08	[0.97, 1.20]	0.0542	1.3793	0.1678	68.9%	35.3389, *p* = 0.0013	1.14	[1.07, 1.21]	0.0293	44.072	**<0.0001**
Verbal learning	1.29	[1.15, 1.45]	0.0589	4.3337	**<0.0001**	77.59%	63.5687, *p* < 0.0001	1.45	[1.29, 1.62]	0.0583	63.549	**<0.0001**
Visual learning	1.20	[1.07, 1.34]	0.0585	3.0629	**0.0022**	68.61%	63.5687, *p* < 0.0001	1.3	[1.14, 1.48]	0.0658	40.036	**<0.0001**
Reasoning and problem solving	1.17	[1.03, 1.34]	0.0673	2.3604	**0.0183**	44.72%	4.8746, *p* = 0.1812	1.17	[1.03, 1.34]	0.0673	23.604	**0.0183**
Social cognition	1.13	[1.00, 1.28]	0.0628	1.9294	0.0537	53.89%	21.3436, *p* = 0.0188	1.19	[1.03, 1.37]	0.0724	23.714	**0.0177**
General intelligence	0.83	[0.56, 1.21]	0.1956	−0.9765	0.3288	97.84%	743.9544, *p* < 0.0001	0.87	[0.59, 1.27]	0.1968	−0.7317	0.4644
Premorbid IQ	1.27	[1.09, 1.49]	0.0794	3.0287	**0.0025**	69.60%	38.2842, *p* < 0.0001	1.33	[1.11, 1.58]	0.0896	31.400	**0.0017**
Visuospatial ability	1.07	[0.99, 1.16]	0.0407	1.7120	0.0869	0	2.2982, *p* = 0.6811	1.17	[1.08, 1.27]	0.0414	38.782	**0.0001**
Verbal memory	1.35	[0.84, 2.19]	0.2454	1.2288	0.2191	94.08%	39.0902, *p* < 0.0001	1.52	[0.90, 2.57]	0.2686	15.567	0.1196
Visual memory	1.41	[1.02, 1.94]	0.1644	2.0831	**0.0372**	84.67%	23.4478, *p* = 0.0003	2.27	[1.00, 5.13]	0.4162	19.675	**0.0491**
Executive functioning	1.31	[1.18, 1.45]	0.0523	5.1875	**<0.0001**	73.55%	100.9611, *p* < 0.000	1.15	[1.01, 1.31]	0.0671	2.0361	**0.0417**
Motor functioning	1.05	[0.90, 1.22]	0.0767	0.6541	0.5130	0	2.1967, *p* = 0.5326	1.09	[0.93, 1.27]	0.0783	10.633	0.2876
Olfaction	0.98	[0.82, 1.17]	0.0922	−0.2533	0.8000	63.43%	12.2393, *p* = 0.0157	1.05	[0.88, 1.26]	0.0901	0.5604	0.5752
**CHR-P developing psychosis and CHR-P not developing psychosis**												
Processing speed	0.97	[0.85, 1.10]	0.0655	−0.4724	0.6366	99.4%	1927.9720, *p* < 0.0001	0.83	[0.64, 1.09]	0.1369	−1.3539	0.1758
Attention/vigilance	0.98	[0.88, 1.10]	0.0579	−0.2913	0.7709	0	4.4449, *p* = 0.3491	1.01	[0.77, 1.35]	0.1438	0.1022	0.9186
Working memory	1.08	[0.76, 1.54]	0.1808	0.4166	0.6770	74.03%	13.1200, *p* = 0.0107	1.02	[0.71, 1.46]	0.1836	0.0831	0.9338
Verbal learning	1.23	[0.87, 1.74]	0.1755	1.1843	0.2363	0	0.3384, *p* = 0.8443	1.23	[0.87, 1.74]	0.1755	11.843	0.2363
General intelligence	0.97	[0.85, 1.10]	0.0643	−0.5167	0.6053	26.34%	7.7246, *p* = 0.3575	0.97	[0.85, 1.1]	0.0643	−0.5167	0.6053
Premorbid IQ	1.23	[0.87, 1.74]	0.1755	1.1843	0.2363	0	0.3384, *p* = 0.8443	1.45	[0.74, 2.81]	0.3398	10.840	0.2783
Visual memory	0.81	[0.61, 1.07]	0.1442	−1.4792	0.1391	37.03%	3.3496, *p* = 0.1873	0.81	[0.61, 1.07]	0.1442	−1.4792	0.1391
Executive functioning	1.38	[1.07, 1.78]	0.1297	2.4934	**0.012**	63.18%	0.7605, *p* = 0.0447	1.38	[1.07, 1.78]	0.1297	24.934	**0.0127**
Motor functioning	1.04	[0.78, 1.38]	0.1447	0.2531	0.8002	0	0.7630, *p* = 0.6829	1.04	[0.78, 1.38]	0.1447	0.2531	0.8002
Olfaction	1.12	[0.81, 1.57]	0.1685	0.6989	0.4846	73.86%	9.6164, *p* = 0.0221	1.12	[0.81, 1.57]	0.1685	0.6989	0.4846
**CHR-P vs FEP**												
Processing speed	1.45	[0.93, 2.28]	0.2281	1.6429	0.1004	87.24%	17.2473, *p* = 0.0002	0.86	[0.57, 1.30]	0.2095	−0.701	0.4832
Verbal learning	1.12	[1.00, 1.26]	0.0591	1.8877	0.0591	0	1.1899, *p* = 0.9458	1.23	[1.09, 1.39]	0.0614	33.450	**0.0008**
General intelligence	1.01	[0.79, 1.28]	0.1231	0.0422	0.9663	42.36%	3.5567, *p* = 0.1689	1.12	[0.90, 1.39]	0.1096	10.275	0.3042
Premorbid IQ	0.80	[0.59, 1.09]	0.1570	−1.3988	0.1619	49.02%	3.9242, *p* = 0.1406	0.79	[0.56, 1.11]	0.1768	−1.3469	0.1780
Executive functioning	1.28	[1.08, 1.51]	0.0861	2.8308	**0.0046**	62.86%	15.4049, *p* = 0.0173	0.88	[0.69, 1.13]	0.1280	−0.9919	0.3212

Bold values identify statistical significance (*p* < 0.05).

**Fig. 2:**
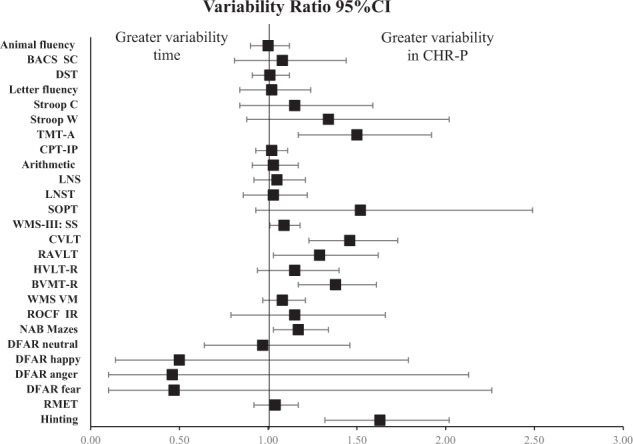
Variability ratio (VR) of neurocognitive functioning between CHR-P vs HC. CHR-P Clinical high risk for Psychosis, HC Healthy Controls, BACS SC indicates Brief Assessment of Cognition Scale Symbol Coding, DST digit symbol coding test, Stroop C Stroop color naming task, StroopW Stroop color word reading task, TMT-A Trail Making Test-Part A, CPT-IP Continuous Performance Test–Identical Pairs, LNS Letter Number Span, LNST Letter Number Sequencing Test, SOPT Self-ordered Pointing Test, WMS-III: SS Wechsler Memory Scale III: Spatial Span, CVLT California Verbal Learning Test, RAVLT Rey Auditory Verbal Learning Test, HVLT-R Hopkins Verbal Learning Test-Revised, BVMT-R Brief Visuospatial Memory Test-Revised, WMS VM Wechsler Memory Scale Immediate Visual Memory, ROCF Rey-Osterrieth Complex Figure Immediate Recall, NAB Mazes Neuropsychological Assessment Battery Mazes, DFAR Degraded Facial Affect Recognition, RMET Reading the Mind in the Eyes Test.

**Fig. 3:**
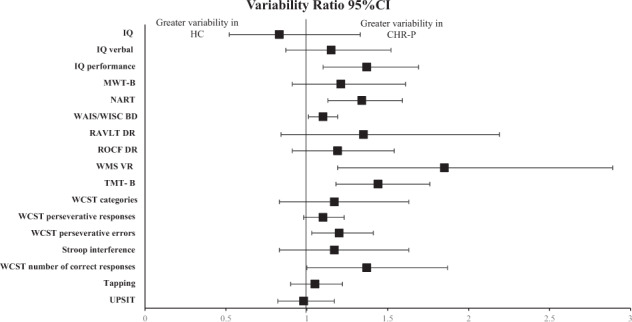
Variability ratio (VR) of neurocognitive functioning between CHR-P vs HC. CHR-P Clinical high risk for Psychosis, HC Healthy Controls, IQ Wechsler Intelligence Scales full, IQ verbal Wechsler Intelligence Scales verbal, IQ performance Wechsler Intelligence Scales performance, NART National Adult Reading Test, MWT-B Mehrfach-Wortschaftz-Intelligenz Test-Part B, RAVLT DR Rey Auditory Verbal Learning Test Delayed Recall, ROCF DR Rey- Osterrieth Complex Figure Test Delayed Recall, WMS VR Weschler Memory Scale Visual Reproduction Delayed Recall, TMT-B Trail Making Test-Part B, WCST categories Wisconsin Card Sorting Test categories, WCST number of correct responses Wisconsin Card Sorting Test number of correct responses, WCST perseverative errors Wisconsin Card Sorting Test perseverative errors, WCST perseverative responses Wisconsin Card Sorting Test perseverative responses, UPSIT University of Pennsylvania Smell Identification Test.

The individual VR according to different tasks is reported in Figs. 2–3. The CHR group presented greater variability than HC in Wechsler Memory Scale Immediate (WMS) Visual Memory (VR: 1.85, 95% CI 1.19–2.89); Hinting task (VR:1.63, 95% CI 1.32–2.02); Trail Making Test-A (TMT-A) (VR: 1.5, 95% CI 1.17–1.92); California Verbal Learning Test (CVLT) (VR: 1.46, 95% CI 1.23–1.73); Trail Making Test-B (TMT-B) (VR: 1.44, 95% CI 1.18–1.76), Brief Visuospatial Memory Test-Revised (BVMT-R) (VR: 1.38, 95% CI 1.17, 1.61), IQ performance (VR: 1.37, 95% CI 1.10–1.69), Wisconsin Card Sort Test (WCST) number of correct responses (VR: 1.37, 95% CI 1.00–1.87); National Adult Reading Test (NART) (VR: 1.34, 95% CI 1.13, 1.59), Rey Auditory Verbal Learning Test (RAVLT) (VR:1.29, 95% CI 1.03–1.62), WCST perseverative errors (VR: 1.2, 95% CI 1.03–1.41), Neuropsychological Assessment Battery Mazes (NAB Mazes) (VR:1.17, 95% CI 1.03–1.34); Wechsler Adult Intelligence Scale/ Wechsler Intelligence Scale for Children Block Design (WAIS/WISC BD) (VR: 1.1, 95% CI 1.01–1.19); WMS-III: SS (VR: 1.09, 95% CI 1.01, 1.18).

**Fig. 4:**
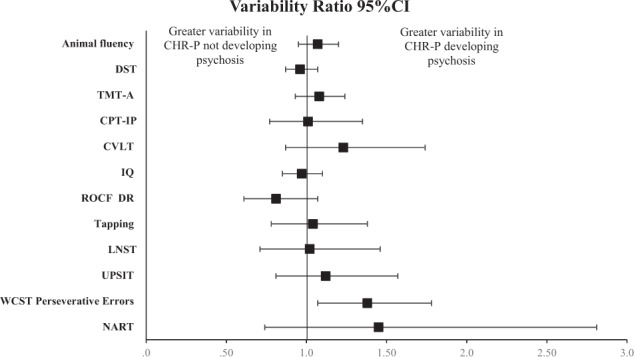
Variability ratio (VR) of neurocognitive functioning between CHR-P transitioning to psychosis vs CHR-P not transitioning to psychosis. CHR-P Clinical high risk for Psychosis, DST Digit Symbol Coding Test, TMT-A Trail Making Test-Part A, CPT Continuous Performance Test-Identical Pairs, CVLT California Verbal Learning Test, IQ Wechsler Intelligence Scales full, ROCF DR Rey-Osterrieth Complex Figure Test Delayed Recall, LNST Letter Number Sequencing Test, UPSIT University of Pennsylvania Smell Identification Test, WCST perseverative errors Wisconsin Card Sorting Test perseverative errors, NART National Adult Reading Test.

**Fig. 5:**
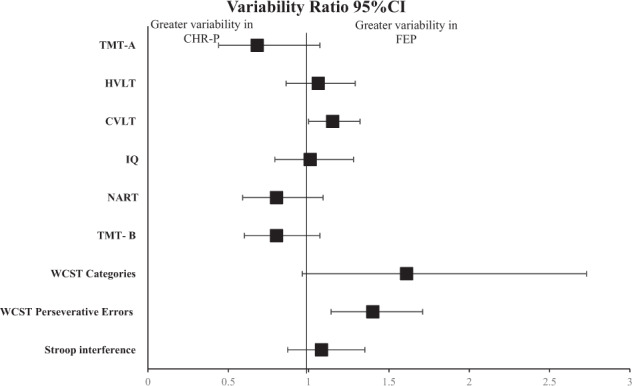
Variability ratio (VR) of neurocognitive functioning between CHR-P vs FEP. CHR-P Clinical high risk for Psychosis, FEP First Episode Psychosis, TMT-A Trail Making Test Part A, HVLT-R Hopkins Verbal Learning Test-Revised, CVLT California Verbal Learning Test, IQ Wechsler Intelligence Scales full, NART National Adult Reading Test, TMT-B Trail Making Test-Part B, WCST categories Wisconsin Card Sorting Test categories, WCST perseverative errors Wisconsin Card Sorting Test perseverative errors.

In CVR analyses, the following tasks showed greater variability in the CHR-P group: Digit Symbol Coding Test (DST) (CVR: 1.14, 95% CI 1.03–1.27), Stroop Color word reading test (CVR: 1.38, 95% CI 1.03–1.85), Stroop Word reading test (CVR: 1.71, 95% CI 1.23–2.37), TMT-A (CVR: 1.31, 95% CI 1.03–1.66), Continuous Performance Test Identical Pairs (CPT-IP) (CVR: 1.19, 95% CI 1.06–1.32), Letter Number Span (LNS) (CVR: 1.18, 95% CI 1.09–1.29), Letter Naming Sequencing Test (LNST) (CVR: 1.15, 95% CI 1.01–1.31), WMS-III: Spatial Span (CVR: 1.19, 95% CI 1.10–1.29), CVLT (CVR: 1.60, 95% CI 1.35–1.91), RAVLT (CVR: 1.41, 95% CI 1.09–1.82), Hopkins Verbal Learning Test-Revised (HVLT-R) (CVR: 1.36, 95% CI 1.14–1.61), BVMT-R (Brief Visuospatial Memory Test-Revised) (CVR: 1.52, 95% CI 1.28–1.80), WMS Visual Memory (CVR: 1.18, 95% CI 1.05–1.32), Neuropsychological Assessment Battery Mazes (CVR: 1.33, 95% CI 1.09–1.62), Hinting (CVR: 1.74, 95% CI 1.39–2.18), IQ performance (CVR: 1.74, 95% CI 1.39–2.18), NART (CVR: 1.40, 95% CI 1.13–1.73), WAIS/WISC BD (CVR: 1.21, 95% CI 1.11–1.31), WMS Visual Reproduction (CVR: 2.55, 95% CI 1.06–6.14), Wisconsin Card Sorting Test (WCST) categories (CVR: 1.28, 95% CI 1.06–1.54), (eTable 6).

### VR and CVR variability of neurocognitive functioning: CHR-P transitioning to psychosis vs CHR-P non-transitioning

The pooled VR neurocognitive domains (Table 1) showed that the CHR-P transitioned group presented greater variability than CHR-P non-transitioned in executive functioning VR: 1.38, 95% CI 1.07, 1.78. In the case of CVR this result remained significant (CVR: 1.38, 95% CI 1.07, 1.78).

The VR between CHR-P developing psychosis and CHR-P not developing psychosis is reported in Fig. 4. The VR was higher for CHR-P transitioned group in WCST perseverative errors (VR: 1.38, 95% CI 1.07–1.78). This result was also significant (CVR: 1.21, 95% CI 1.01–1.46) in CVR analyses; moreover, animal fluency (CVR: 1.19, 95% CI 1.01–1.4) showed also greater variability in CHR-P transitioned.

### VR and CVR variability of neurocognitive functioning: CHR-P vs FEP

The pooled VR neurocognitive domains (Table 1) demonstrated that the FEP group presented greater variability than CHR-P in executive functioning (VR: 1.28, 95% CI 1.08, 1.51). CVR analyses presented a greater variability for the FEP group in verbal learning (CVR: 1.23, 95% CI 1.09, 1.39).

The VR between CHR-P and FEP is reported in Fig. 5. This VR variability was higher in WCST Perseverative Errors (VR: 1.03, 95% CI 1.4, 1.14,) while CVR presented greater variability in CVLT (CVR: 1.24, 95% CI 1.07–1.44), and WCST categories (CVR: 1.81, 95% CI 1.18–2.79).

### Study quality, heterogeneity and metaregressions

The quality rating of the studies ranged from 4 to 8 (average = 5.8; median = 6), see eTable 5. Heterogeneity ranged from small 0% (DST, arithmetic, WMS-III SS, VMS VM, RMET, and tapping tasks) to large 98.68% (DFAR fear task) in CHR-P vs HC comparisons, from 0% (animal fluency, CVLT, TMT-A, and tapping tasks) to 81.74% (NART) task) in CHR-P not transitioning vs CHR-P transitioning, and from 0% (HVLT) to 91.84% (WCST categories task) in CHR-P vs FEP groups.

Regarding metaregressions, in the CHR-P vs HC analyses, higher age (β = −0.06, SE = 0.02, *p* = 0.007) was associated with reduced variability of processing speed. Any other metaregression (age, sex, years of education, race, functioning, attenuated psychotic symptoms, NOS) was not significant (see eTable 7–9).

## Discussion

We have presented the first large-scale meta-analytic comparative investigation of within-group neurocognitive performance variability in a help-seeking CHR-P population, compared to HC and FEP. Our first main finding is that the within-studies variability was greater in several neurocognitive domains (visual memory, executive functioning, verbal learning, premorbid IQ, processing speed, visual learning, and reasoning and problem solving) in the CHR-P state with respect to HC using both the VR and CVR metrics. Besides, the CVR analyses demonstrated also greater variability in attention/vigilance, working memory, social cognition, and visuospatial ability.

This is the first meta-analysis investigating the variability of neurocognitive profiles in the CHR-P population compared with HC. Importantly, the CVR analysis confirmed increased variability in the very same domains. This greater variability in CHR-P was a highly robust finding, as it was confirmed by two variability measurements, thus improving the confidence with which we can infer that the variability is strengthened by meaningful neurocognitive mechanisms as opposed to confounding factors. Recent work [54] identified a two-cluster solution comprising a neurocognitively non-impaired and a neurocognitively impaired subgroup in a combined sample of both CHR-P and HC and found that the impaired subgroup was more prevalent in the CHR-P population, confirming that the neurocognitive profile of CHR-P may vary broadly [54]. This study moves beyond the between-study variance reported in our prior paper (such as with the I^2^ statistic) [8], by showing that CHR-P individuals had higher VR/CVR variability in several neurocognitive domains compared to HC. This indicates that the average measurement of neurocognitive functioning may not represent a solid estimate in this group. Overall, these findings call for future research deconstructing the demonstrated variability of neurocognitive functioning at the individual subject level. Individual-participant data meta-analyses may prove to be particularly useful, as well as precision medicine approaches that leverage neurocognitive domains [55, 56].

To address these questions, we also demonstrated that the specific neurocognitive tasks affected by high variability (in VR and CVR analyses) involve WMS Visual Memory, Hinting task, TMT-A, CVLT, BVMT-R, NART, RAVLT, NAB Mazes, WAIS/WISC BD, WMS-III: SS in CHR-P population. Several predicting psychosis models, which include neurocognitive domains, in CHR-P has been proposed [57–64]. These models are based on previous literature about this topic in this population. This study could help select the most valuable tasks for these prediction models highlighting these tasks with the higher power of finding the subjects more deteriorated prior to the onset of the psychosis.

The present data also investigated whether this variability in neurocognitive profiles may be associated with the heterogeneous clinical outcomes of the CHR-P stage, with only about one-third of them fully remitting from their initial problems [65]. We found that the variability in the neurocognitive performance in CHR-P not developing psychosis was similar to the CHR-P developing psychosis group (except for the perseverative errors task showed higher variability in CHR-P developing psychosis compared to CHR-P not developing psychosis) and the FEP subjects (except for the perseverative errors task showed higher variability in FEP compared to CHR-P), suggesting that neurocognitive variability is equally presented across the two stages of the disorders. Our findings are consistent with a broad literature showing that neurocognitive impairments predate the onset of psychosis and with findings showing limited neurocognitive changes between the latter part of the CHR-P period and the onset of the FEP [66, 67]. We therefore suggest that a majority of neurocognitive variability may occur before psychosis onset. Similarly, longitudinal studies of children who later develop psychoses show lower levels of overall cognitive abilities (IQs) and lower levels of academic achievement [68, 69].

Interpreting these findings altogether is challenging. The observed increased variability in CHR-P could just be the same variability that exists in healthy people—i.e. there is a constant effect. Accordingly, the CHR-P group would represent those from the general population that would have been at the tail end of the bell curve in the healthy population if they had never developed a disorder. However, CHR-P individuals are symptomatic at presentation and not generally representative of the general population because of important sampling biases discussed in the introduction [29]. An alternative hypothesis is that in some CHR-P individuals, the development of a psychotic disorder had a drastic impact on neurocognitive functioning, while it had no/minimal effect in others, thus leading to no VR/CVR differences between CHR-P and FEP.

There are several limitations to consider in this study. Firstly, the validity of the VR and CVR as an index of variability has received debate in the context of other clinical factors, such as individual treatment response and subgroup effects [70]. Second, we have been unable to find variability in the VR across different subgroups of CHR-P (BLIPS, APS, and GRD) because these data were not reliably available in enough studies. This is the first study addressing this topic so future evidence-syntheses approaches in the CHR-P field could consider individual CHR-P subgroups [28]. This is more likely obtained in large-scale prospective cohorts such as ProNET [71], PRESCIENT [72] that are now ongoing. This is also the case in the FEP groups, where there are not enough data to explore the differences in the neurocognitive domains according to the specific designation. Third, whilst the analyses did not show an association with all clinical features, this may be attributable to the low statistical power of our metaregressions. And finally, the focus of future studies may clarify those potential influential factors that could not be studied in terms of their relationship to domain/task variability. Sources of variability in previous neurocognitive studies include premorbid-developmental status, substance exposure, adversity/trauma or poverty during the developmental period. Psychometric features of specific tasks may be also an important contributing factor to these results; presumably, those that have lower reliability would likely yield greater variability. Some of that variable reliability may be associated with examiner effects related to administration and scoring. So, some of the observed heterogeneity may be a function of test features and their administration and scoring. The main aim of precision psychiatry [55, 73] approaches is to embed personalized information into clinical care by generating a comprehensive matrix of information for each patient, based on disease pathophysiology, in order to forecast outcomes or responses to interventions at the individual subject level. Our results suggest a disorder-related variability above the level typically observed in the healthy population. This may rule out a constant effect of CHR-P status on neurocognitive performance, e.g. that there is a uniform reduction of IQ shifting the overall neurocognitive profile of this patient group. Instead, our findings indicate that different CHR-P individuals are affected by variable neurocognitive deficits. These findings strengthen the implication of precision psychiatry approaches: an increased variability requires personalizing preventive interventions in this group. The detection of these specific subgroups according to their neurocognitive profile provides evidence to personalize the interventions; this study may represent the starting point for future interventional research in this area.

Although an increased neurocognitive variability in CHR-P individuals compared to controls was to be expected based on their underlying heterogeneity, this is the first study to have empirically demonstrated it for the neurocognitive domain. Furthermore, before this study it was not clear which specific neurocognitive domain was more or less affected by increased variability. We demonstrated that the variability was greater in several neurocognitive domains (visual memory, executive functioning, verbal learning, premorbid IQ, processing speed, visual learning, and reasoning and problem solving) in the CHR-P state with respect to HC.

In conclusions, our findings suggest the existence of significant variability with different neurocognitive profiles of CHR-P groups, suggesting that the average measurement of neurocognitive functioning may not represent a solid estimate in this group. This finding should be explored by further individual-participant data research and support precision medicine approaches.

## Supplementary information

SUPPLEMENTAL MATERIAL

## References

[1] Sheffield JM, Karcher NR, Barch DM (2018). Cognitive deficits in psychotic disorders: a lifespan perspective. Neuropsychol Rev..

[2] Aas M, Dazzan P, Mondelli V, Melle I, Murray RM, Pariante CM (2014). A systematic review of cognitive function in first-episode psychosis, including a discussion on childhood trauma, stress, and inflammation. Front Psychiatry.

[3] Carruthers SP, Van Rheenen TE, Gurvich C, Sumner PJ, Rossell SL (2019). Characterising the structure of cognitive heterogeneity in schizophrenia spectrum disorders. A systematic review and narrative synthesis. Neurosci Biobehav Rev..

[4] Heilbronner U, Samara M, Leucht S, Falkai P, Schulze TG (2016). The longitudinal course of schizophrenia across the lifespan: clinical, cognitive, and neurobiological aspects. Harv Rev Psychiatry.

[5] Green MF, Horan WP, Lee J (2019). Nonsocial and social cognition in schizophrenia: current evidence and future directions. World Psychiatry.

[6] Stone WS, Seidman LJ. Developmental Psychopathology. 3rd edn. John Wiley & Sons; 2016.

[7] Woodberry KA, Giuliano AJ, Seidman LJ (2008). Premorbid IQ in schizophrenia: a meta-analytic review. Am J Psychiatry.

[8] Catalan A, Salazar de Pablo G, Aymerich C, Damiani S, Sordi V, Radua J, et al. Neurocognitive functioning in individuals at clinical high risk for psychosis: A systematic review and meta-analysis. JAMA Psychiatry. 2021:e211290. 10.1001/jamapsychiatry.2021.1290. (Epub ahead of print). 10.1001/jamapsychiatry.2021.1290 PMC8209603 34132736

[9] Salazar de Pablo G, Radua J, Pereira J, Bonoldi I, Arienti V, Besana F (2021). Probability of transition to psychosis in individuals at clinical high risk: an updated meta-analysis. JAMA Psychiatry.

[10] Catalan A, Salazar de Pablo G, Vaquerizo Serrano J, Mosillo P, Baldwin H, Fernandez-Rivas A, et al. Annual Research Review: Prevention of psychosis in adolescents - systematic review and meta-analysis of advances in detection, prognosis and intervention. J Child Psychol Psychiatry. 2021;62:657–73. 10.1111/jcpp.13322. 10.1111/jcpp.13322 32924144

[11] Salazar de Pablo G, Catalan A, Fusar-Poli P (2020). Clinical validity of DSM-5 attenuated psychosis syndrome: advances in diagnosis, prognosis, and treatment. JAMA Psychiatry.

[12] Seidman LJ, Shapiro DI, Stone WS, Woodberry KA, Ronzio A, Cornblatt BA (2016). Association of neurocognition with transition to psychosis: baseline functioning in the second phase of the north american prodrome longitudinal study. JAMA Psychiatry.

[13] Bechi M, Bosia M, Spangaro M, Buonocore M, Cavedoni S, Agostoni G (2017). Exploring functioning in schizophrenia: predictors of functional capacity and real-world behaviour. Psychiatry Res..

[14] Fett AJ, Velthorst E, Reichenberg A, Ruggero CJ, Callahan JL, Fochtmann LJ (2020). Long-term changes in cognitive functioning in individuals with psychotic disorders: findings from the Suffolk County Mental Health Project. JAMA Psychiatry.

[15] Mahmood Z, Burton CZ, Vella L, Twamley EW (2018). Neuropsychological predictors of performance-based measures of functional capacity and social skills in individuals with severe mental illness. J Psychiatr Res..

[16] Rempfer MV, Fowler CA (2018). Relationships among functional capacity, cognition, and naturalistic skill performance in people with serious mental illness. Psychiatry Res..

[17] Feczko E, Miranda-Dominguez O, Marr M, Graham AM, Nigg JT, Fair DA (2019). The heterogeneity problem: approaches to identify psychiatric subtypes. Trends Cogn Sci..

[18] Green MJ, Girshkin L, Kremerskothen K, Watkeys O, Quide Y (2020). A systematic review of studies reporting data-driven cognitive subtypes across the psychosis spectrum. Neuropsychol Rev..

[19] Rabelo-da-Ponte FD, Lima FM, Martinez-Aran A, Kapczinski F, Vieta E, Rosa AR, et al. Data-driven cognitive phenotypes in subjects with bipolar disorder and their clinical markers of severity. Psychol Med. 2020:1–8. 10.1017/S0033291720003499. (Epub ahead of print). 10.1017/S0033291720003499 33050962

[20] Lee J, Rizzo S, Altshuler L, Glahn DC, Miklowitz DJ, Sugar CA (2017). Deconstructing Bipolar Disorder and Schizophrenia: a cross-diagnostic cluster analysis of cognitive phenotypes. J Affect Disord.

[21] Lee RS, Hermens DF, Naismith SL, Lagopoulos J, Jones A, Scott J (2015). Neuropsychological and functional outcomes in recent-onset major depression, bipolar disorder and schizophrenia-spectrum disorders: a longitudinal cohort study. Transl Psychiatry.

[22] Lewandowski KE, Baker JT, McCarthy JM, Norris LA, Ongur D (2018). Reproducibility of cognitive profiles in psychosis using cluster analysis. J Int Neuropsychol Soc.

[23] Van Rheenen TE, Lewandowski KE, Tan EJ, Ospina LH, Ongur D, Neill E (2017). Characterizing cognitive heterogeneity on the schizophrenia-bipolar disorder spectrum. Psychol Med..

[24] Fusar-Poli P, Cappucciati M, Borgwardt S, Woods SW, Addington J, Nelson B (2016). Heterogeneity of psychosis risk within individuals at clinical high risk: a meta-analytical stratification. JAMA Psychiatry.

[25] Fusar-Poli P, Cappucciati M, Bonoldi I, Hui LM, Rutigliano G, Stahl DR (2016). Prognosis of brief psychotic episodes: a meta-analysis. JAMA Psychiatry.

[26] Fusar-Poli P, Cappucciati M, De Micheli A, Rutigliano G, Bonoldi I, Tognin S (2017). Diagnostic and prognostic significance of brief limited intermittent psychotic symptoms (BLIPS) in individuals at ultra high risk. Schizophr Bull.

[27] Fusar-Poli P, De Micheli A, Chalambrides M, Singh A, Augusto C, McGuire P (2019). Unmet needs for treatment in 102 individuals with brief and limited intermittent psychotic symptoms (BLIPS): implications for current clinical recommendations. Epidemiol Psychiatr Sci.

[28] Fusar-Poli P, Salazar de Pablo G, Rajkumar RP, López-Díaz A, Malhotra S, Heckers S, et al. Diagnosis, prognosis, and treatment of Brief Psychotic Episodes: a review and research agenda Lancet Psychiatr. 2022;9:72–83. 10.1016/S2215-0366(21)00121-8. 10.1016/S2215-0366(21)00121-8 34856200

[29] Fusar-Poli P, Schultze-Lutter F, Cappucciati M, Rutigliano G, Bonoldi I, Stahl D (2016). The Dark side of the moon: meta-analytical impact of recruitment strategies on risk enrichment in the clinical high risk state for psychosis. Schizophr Bull..

[30] Fusar-Poli P, Rutigliano G, Stahl D, Schmidt A, Ramella-Cravaro V, Hitesh S (2016). Deconstructing pretest risk enrichment to optimize prediction of psychosis in individuals at clinical high risk. JAMA Psychiatry.

[31] Fusar-Poli P, Tantardini M, De Simone S, Ramella-Cravaro V, Oliver D, Kingdon J (2017). Deconstructing vulnerability for psychosis: meta-analysis of environmental risk factors for psychosis in subjects at ultra high-risk. Eur Psychiatry..

[32] McGorry PD (2013). Early clinical phenotypes, clinical staging, and strategic biomarker research: building blocks for personalized psychiatry. Biol Psychiatry.

[33] Addington J, Farris M, Devoe D, Metzak P (2020). Progression from being at-risk to psychosis: next steps. npj Schizophr..

[34] Tognin S, van Hell HH, Merritt K, Winter-van Rossum I, Bossong MG, Kempton MJ (2020). Towards precision medicine in psychosis: benefits and challenges of multimodal multicenter studies-PSYSCAN: translating neuroimaging findings from research into clinical practice. Schizophr Bull.

[35] Hauser M, Zhang J-P, Sheridan EM, Burdick KE, Mogil R, Kane JM (2017). Neuropsychological test performance to enhance identification of subjects at clinical high risk for psychosis and to be most promising for predictive algorithms for conversion to psychosis: a meta-analysis. J Clin Psychiatry.

[36] Fusar-Poli P, Deste G, Smieskova R, Barlati S, Yung AR, Howes O (2012). Cognitive Functioning in prodromal psychosis a meta-analysis. Arch Gen Psychiatry.

[37] Pillinger T, Osimo EF, Brugger S, Mondelli V, McCutcheon RA, Howes OD (2019). A Meta-analysis of immune parameters, variability, and assessment of modal distribution in psychosis and test of the immune subgroup hypothesis. Schizophr Bull.

[38] McCutcheon RA, Jauhar S, Pepper F, Nour MM, Rogdaki M, Veronese M (2020). The topography of striatal dopamine and symptoms in psychosis: an integrative positron emission tomography and magnetic resonance imaging study. Biol Psychiatry Cogn Neurosci Neuroimaging.

[39] Rogdaki M, Gudbrandsen M, McCutcheon RA, Blackmore CE, Brugger S, Ecker C (2020). Magnitude and heterogeneity of brain structural abnormalities in 22q11.2 deletion syndrome: a meta-analysis. Mol Psychiatry.

[40] Guo X, McCutcheon RA, Pillinger T, Mizuno Y, Natesan S, Brown K (2020). The magnitude and heterogeneity of antidepressant response in depression: a meta-analysis of over 45,000 patients. J Affect Disord.

[41] Maslej MM, Furukawa TA, Cipriani A, Andrews PW, Sanches M, Tomlinson A (2021). Individual differences in response to antidepressants: a meta-analysis of placebo-controlled randomized clinical trials. JAMA Psychiatry.

[42] Mizuno Y, McCutcheon RA, Brugger SP, Howes OD (2020). Heterogeneity and efficacy of antipsychotic treatment for schizophrenia with or without treatment resistance: a meta-analysis. Neuropsychopharmacology..

[43] McCutcheon RA, Pillinger T, Mizuno Y, Montgomery A, Pandian H, Vano L (2021). The efficacy and heterogeneity of antipsychotic response in schizophrenia: a meta-analysis. Mol Psychiatry.

[44] Meyer N, Faulkner SM, McCutcheon RA, Pillinger T, Dijk DJ, MacCabe JH. Sleep and circadian rhythm disturbance in remitted schizophrenia and bipolar disorder: a systematic review and meta-analysis. Schizophr Bull. 2020;46:1126–43. 10.1093/schbul/sbaa024. 10.1093/schbul/sbaa024 PMC7505194 32154882

[45] Radua J, Davies C, Fusar-Poli P (2021). Evaluation of variability in individual response to treatments in the clinical high-risk state for psychosis: a meta-analysis. Schizophr Res..

[46] Winkelbeiner S, Leucht S, Kane JM, Homan P (2019). Evaluation of differences in individual treatment response in schizophrenia spectrum disorders: a meta-analysis. JAMA Psychiatry..

[47] Brugger SP, Howes OD (2017). Heterogeneity and homogeneity of regional brain structure in schizophrenia: a meta-analysis. JAMA Psychiatry..

[48] Moher D, Liberati A, Tetzlaff J, Altman DG, Group P (2009). Preferred reporting items for systematic reviews and meta-analyses: the PRISMA statement. J Clin Epidemiol..

[49] Stroup DF, Berlin JA, Morton SC, Olkin I, Williamson GD, Rennie D (2000). Meta-analysis of observational studies in epidemiology: a proposal for reporting. Meta-analysis Of Observational Studies in Epidemiology (MOOSE) group. JAMA..

[50] Altman DG, Simera I, Hoey J, Moher D, Schulz K (2008). EQUATOR: reporting guidelines for health research. Lancet..

[51] Senior AM, Viechtbauer W, Nakagawa S (2020). Revisiting and expanding the meta-analysis of variation: the log coefficient of variation ratio. Res Synth Methods..

[52] Team RC. R: A language and environment for statistical computing. 1.4.1106 ed. Vienna, Austria: R Foundation for Statistical Computing; 2021.

[53] Viechtbauer W. The Comprehensive R Archive Network. Package ‘Metafor’. 2015. https://cran.r-project.org/web/packages/metafor/index.html.

[54] Haining K, Gajwani R, Gross J, Gumley AI, Ince RAA, Lawrie SM, et al. Characterising cognitive heterogeneity in individuals at clinical high-risk for psychosis: a cluster analysis with clinical and functional outcome prediction. Eur Arch Psychiatr Clin Neurosci. 2022;272:437–48. 10.1007/s00406-021-01315-2. 10.1007/s00406-021-01315-2 PMC8938352 34401957

[55] Salazar de Pablo G, Studerus E, Vaquerizo-Serrano J, Irving J, Catalan A, Oliver D, et al. Implementing precision psychiatry: a systematic review of individualized prediction models for clinical practice. Schizophr Bull. 2021;47:284–97. 10.1093/schbul/sbaa120. 10.1093/schbul/sbaa120 PMC7965077 32914178

[56] Koutsouleris N, Worthington M, Dwyer DB, Kambeitz-Ilankovic L, Sanfelici R, Fusar-Poli P (2021). Toward generalizable and transdiagnostic tools for psychosis prediction: an independent validation and improvement of the NAPLS-2 risk calculator in the multisite PRONIA cohort. Biol Psychiatry.

[57] Ziermans T, de Wit S, Schothorst P, Sprong M, van Engeland H, Kahn R (2014). Neurocognitive and clinical predictors of long-term outcome in adolescents at ultra-high risk for psychosis: a 6-year follow-up. PLoS ONE..

[58] Koutsouleris N, Dwyer DB, Degenhardt F, Maj C, Urquijo-Castro MF, Sanfelici R (2021). Multimodal machine learning workflows for prediction of psychosis in patients with clinical high-risk syndromes and recent-onset depression. JAMA Psychiatry..

[59] Riecher-Roessler A, Pflueger MO, Aston J, Borgwardt SJ, Brewer WJ, Gschwandtner U (2009). Efficacy of using cognitive status in predicting psychosis: a 7-year follow-up. Biol Psychiatry..

[60] Pukrop R, Ruhrmann S, Schultze-Lutter F, Bechdolf A, Brockhaus-Dumke A, Klosterkotter J (2007). Neurocognitive indicators for a conversion to psychosis: comparison of patients in a potentially initial prodromal state who did or did not convert to a psychosis. Schizophr Res.

[61] Lencz T, Smith CW, McLaughlin D, Auther A, Nakayama E, Hovey L (2006). Generalized and specific neurocognitive deficits in prodromal schizophrenia. Biol Psychiatry..

[62] Cornblatt BA, Carrion RE, Auther A, McLaughlin D, Olsen RH, John M (2015). Psychosis prevention: a modified clinical high risk perspective from the recognition and prevention (RAP) program. Am J Psychiatry..

[63] Corcoran CM, Keilp JG, Kayser J, Klim C, Butler PD, Bruder GE (2015). Emotion recognition deficits as predictors of transition in individuals at clinical high risk for schizophrenia: a neurodevelopmental perspective. Psychol Med.

[64] Mittal VA, Walker EF, Bearden CE, Walder D, Trottman H, Daley M (2010). Markers of basal ganglia dysfunction and conversion to psychosis: neurocognitive deficits and dyskinesias in the prodromal period. Biol Psychiatry..

[65] Salazar de Pablo G, Besana F, Arienti V, Catalan A, Vaquerizo-Serrano J, Cabras A (2021). Longitudinal outcome of attenuated positive symptoms, negative symptoms, functioning and remission in people at clinical high risk for psychosis: a meta-analysis. EClinicalMedicine..

[66] Bora E, Murray RM (2014). Meta-analysis of cognitive deficits in ultra-high risk to psychosis and first-episode psychosis: do the cognitive deficits progress over, or after, the onset of psychosis?. Schizophr Bull.

[67] Carrion RE, Walder DJ, Auther AM, McLaughlin D, Zyla HO, Adelsheim S (2018). From the psychosis prodrome to the first-episode of psychosis: no evidence of a cognitive decline. J Psychiatr Res..

[68] Mollon J, Reichenberg A (2018). Cognitive development prior to onset of psychosis. Psychol Med..

[69] Kremen WS, Vinogradov S, Poole JH, Schaefer CA, Deicken RF, Factor-Litvak P (2010). Cognitive decline in schizophrenia from childhood to midlife: a 33-year longitudinal birth cohort study. Schizophr Res..

[70] Bae S (2020). Is variance ratio a valid indicator of heterogeneous treatment effect?. JAMA Psychiatry..

[71] Washington University School of Medicine in St. Louis. Psychosis-Risk Outcomes Network (ProNET). 2020. https://werc.wustl.edu/Research/ProNET.

[72] University of Melbourne. Trajectories and predictors in the Clinical High Risk for Psychosis population: Prediction Scientific Global Consortium (PRESCIENT). 2022. https://taggs.hhs.gov/Detail/AwardDetail?arg_AwardNum=U01MH124631&arg_ProgOfficeCode=134.

[73] Fusar-Poli P, Hijazi Z, Stahl D, Steyerberg EW (2018). The science of prognosis in psychiatry: a review. JAMA Psychiatry..

