# Supplementary material for: Examining the variability of neurocognitive functioning in individuals at clinical high risk for psychosis: a meta-analysis
Source: Transl Psychiatry. 2022 May 12;12:198. doi: 10.1038/s41398-022-01961-7 (PMC9098884; doi:10.1038/s41398-022-01961-7)
Supplement: Supplementary file 1 — SUPPLEMENTAL MATERIAL [file 41398_2022_1961_MOESM1_ESM.docx]

Supplementary material

[eTable 1. PRISMA statement and checklist 2](#_Toc101163628)

[eTable 2. MOOSE checklist 4](#_Toc101163629)

[e. Methods 1. Search terms. 6](#_Toc101163630)

[eTable 3. Neurocognitive domains considered in the current meta-analysis (7 MATRICS domains and 8 CHR-P domains. Adapted from Fusar-Poli et al. 2012 ^4^ and Hauser et al. 2017^5^. CHR-P, Clinical High Risk for Psychosis. 6](#_Toc101163631)

[eTable 4. Risk of bias (quality) assessment using modified Newcastle-Ottawa Scale for cross-sectional and cohort studies 7](#_Toc101163632)

[eTable 5. Characteristics of included studies. CHR-P, Clinical High Risk for Psychosis; HC, Healthy Controls; FEP, First Episode Psychosis; FUP, Follow-up; mo, months; NOS, Newcastle-Ottawa Scale. 8](#_Toc101163633)

[eTable 6. Coefficient of Variation Rations (CVR) analyses. 12](#_Toc101163634)

[eTable 7. Metaregressions CHR-P vs HC 15](#_Toc101163635)

[eTable 8. Metaregressions CHR-transitioning vs CHR-non transitioning 17](#_Toc101163636)

[eTable 9. Metaregressions CHR-P vs FEP 17](#_Toc101163637)

# **eTable 1**. PRISMA statement and checklist

| **Section/topic** | **#** | **Checklist item** | ****Page** |
| --- | --- | --- | --- |
| **TITLE** | | |  |
| Title | 1 | Identify the report as a systematic review, meta-analysis, or both | *Cover page* |
| **ABSTRACT** | | | ** |
| Structured summary | 2 | Provide a structured summary including, as applicable: background; objectives; data sources; study eligibility criteria, participants, and interventions; study appraisal and synthesis methods; results; limitations; conclusions and implications of key findings; systematic review registration number | *Abstract* |
| **INTRODUCTION** | | | ** |
| Rationale | 3 | Describe the rationale for the review in the context of what is already known | *Introduction* |
| Objectives | 4 | Provide an explicit statement of questions being addressed with reference to participants, interventions, comparisons, outcomes, and study design (PICOS) | *Introduction* |
| **METHODS** | | | ** |
| Protocol and registration | 5 | Indicate if a review protocol exists, if and where it can be accessed (e.g. Web address), and, if available, provide registration information including registration number | *Methods* |
| Eligibility criteria | 6 | Specify study characteristics (e.g. PICOS length of follow-up) and report characteristics (e.g. years considered, language, publication status) used as criteria for eligibility, giving rationale | *Methods* |
| Information sources | 7 | Describe all information sources (e.g., databases with dates of coverage, contact with study authors to identify additional studies) in the search and date last searched | *Methods* |
| Search | 8 | Present full electronic search strategy for at least one database, including any limits used, such that it could be repeated | *Methods* |
| Study selection | 9 | State the process for selecting studies (i.e. screening, eligibility, included in systematic review, and, if applicable, included in the meta-analysis) | *Methods* |
| Data collection process | 10 | Describe method of data extraction from reports (e.g. piloted forms, independently, in duplicate) and any processes for obtaining and confirming data from investigators | *Methods* |
| Data items | 11 | List and define all variables for which data were sought (e.g. PICOS funding sources) and any assumptions and simplifications made | *Methods* |
| Risk of bias in individual studies | 12 | Describe methods used for assessing risk of bias of individual studies (including specification of whether this was done at the study or outcome level), and how this information is to be used in any data synthesis | *Methods* |
| Summary measures | 13 | State the principal summary measures | *Methods* |
| Risk of bias across studies | 15 | Specify any assessment of risk of bias (i.e. Newcastle-Ottawa Scale (NOS)), that may affect the cumulative evidence | *Methods* |
| Additional analyses | 16 | Describe methods of additional analyses (e.g. sensitivity or subgroup analyses, meta-regression), if done, indicating which were pre-specified | *Methods* |
| **RESULTS** |  |  |  |
| Study selection | 17 | Give numbers of studies screened, assessed for eligibility, and included in the review with reasons for exclusions at each stage, ideally with a flow diagram | *Results* |
| Study characteristics | 18 | For each study, present characteristics for which data were extracted (e.g. study size, PICOS follow-up period) and provide the citations | *Results* |
| Risk of bias within studies | 19 | Present data on risk of bias of each study and, if available, any outcome level assessment (see item 12) | *Results* |
| Results of individual studies | 20 | For all outcomes considered (benefits or harms), present for each study a summary data for each intervention group | *Results* |
| Synthesis of results | 21 | Present results of analyses | *Results* |
| Risk of bias across studies | 22 | Present results of any assessment of the risk of bias across studies (see Item 15) | *Results* |
| Additional analysis | 23 | Give results of additional analyses, if done (e.g. sensitivity or subgroup analyses, meta-regression see Item 16) | *Results* |
| **DISCUSSION** |  |  |  |
| Summary of evidence | 24 | Summarize the main findings including the strength of evidence for each main outcome; consider their relevance to key groups (e.g. healthcare providers, users, and policymakers) | *Discussion* |
| Limitations | 25 | Discuss limitations at study and outcome level (e.g. risk of bias), and at review-level (e.g. incomplete retrieval of identified research, reporting bias) | *Discussion* |
| Conclusions | 26 | Provide a general interpretation of the results in the context of other evidence, and implications for future research | *Discussion* |
| **FUNDING** |  |  |  |
| Funding | 27 | Describe sources of funding for the systematic review and other support (e.g. supply of data), role of funders for the systematic review | *Funding* |

# **eTable 2**. MOOSE checklist

| **Criteria** | | **Brief description of how the criteria were handled in the meta-analysis** |
| --- | --- | --- |
| **Reporting of background should include** | |  |
| √ | Problem definition | To examine at a meta-analytical level whether neurocognitive deficits are evident in Clinical High Risk (CHR-P) for psychosis subjects relative to preferably healthy controls (HC) and to define the specific pattern of these neurocognitive deficits.  To identify neurocognitive impairments that specifically predicted the later transition to psychosis in the CHR-P population, controlling for the potential confounding effect of socio-demographical, methodological, and clinical factors. |
| √ | Hypothesis statement | We hypothesized that CHR-P state would have a significant impairment in neurocognitive domains, especially those who develop psychosis. |
| √ | Description of study outcomes | In line with our earlier meta-analysis the different neurocognitive tasks were grouped in neurocognitive domains on the basis of the criteria developed by the MATRICS conference and then discussed by us, according to the indications of the articles included: (1) processing speed, (2) verbal learning, (3) working memory, (4) reasoning and problem-solving, (5) visual learning, (5) attention and vigilance, and (7) social cognition. Further, we have analysed the CHR-P domains of (8) general intelligence, (9) premorbid intelligence, (10) visuospatial ability, (11) verbal memory, (12) visual memory, (13) executive functioning, (14) motor functioning, and (15) olfaction. We reported differences between CHR-P population and HC in these domains, measured by standardised scales.  For comprehensiveness, we conducted two supplementary meta-analyses: iii) comparing neurocognitive functioning in CHR-P individuals vs FEP individuals (when these contrasts were reported in the articles retrieved) and iv) estimating the pooled effect sizes across each of the 15 neurocognitive domains. For the latter meta-analysis (iv), we followed meta-analytical guidelines(1, 2) to account for studies reporting on more than one non-independent neurocognitive tasks within the same neurocognitive domain. |
| √ | Type of exposure or intervention used | We included individual studies that reported neurocognitive data in CHR-P population. |
| √ | Type of study designs used | Case-control studies, and cohort studies, which investigate the neurocognitive functioning CHR-P for psychosis compared to HC. |
| √ | Study population | CHR-P state. |
| **Reporting of search strategy should include** | |  |
| √ | Qualifications of researchers | The credentials of the investigators are indicated in the author list and in the acknowledgements. |
| √ | Search strategy. including time period included in the synthesis and keywords | We performed a multi-step literature search using the following keywords: "cognit*" OR "neurocognit*" OR "social cognit*" AND "psychosis risk" OR "prodrom*" OR "ultra-high risk" OR "clinical high risk" OR "genetic high risk" OR "at risk mental state" OR "at-risk mental state" OR "basic symptoms" OR “ultra-high risk” from inception until 1st July 2020. |
| √ | Databases and registries searched | Web of Science database (Clarivate Analytics): Web of Science Core Collection, BIOSIS Citation Index, KCI-Korean Journal Database, MEDLINE, Russian Science Citation Index, PubMed and SciELO Citation Index. |
| √ | Use of hand searching | We hand-searched bibliographies of retrieved papers for additional references. |
| √ | List of citations located and those excluded. including justifications | Details of the literature search process are outlined in the results section and in the PRISMA flow-chart. |
| √ | Method of addressing articles published in languages other than English | Only articles in English language were selected. |
| √ | Method of handling abstracts and unpublished studies | Original individual studies were included. Conference proceedings, reviews, editorials, clinical cases and unpublished studies were excluded. |
| √ | Description of any contact with authors | A description of the contact with corresponding authors to request additional data for this study is detailed in methods section. |
| **Reporting of methods should include** | |  |
| √ | Description of relevance or appropriateness of studies assembled for assessing the hypothesis to be tested | Detailed inclusion and exclusion criteria were described in the methods section. |
| √ | Rationale for the selection and coding of data | Data extracted from each of the studies were relevant to the population characteristics, study design, comparison group, exposure and outcomes. |
| √ | Assessment of confounding factors | Confounding factors were systematically assessed in each neurocognitive domain. |
| √ | Assessment of study quality | We adapted the Newcastle-Ottawa Scale for the evaluation of cross-sectional and cohort studies. |
| √ | Assessment of heterogeneity | Heterogeneity was assessed with the I^2^ index. |
| √ | Description of statistical methods in sufficient detail to be replicated | Statistical methods are described in detail in the methods section. |
| √ | Provision of appropriate tables and graphics | We included the PRISMA flow-chart and several tables and graphics to describe the literature search and our results. |
| **Reporting of results should include** | |  |
| √ | Graph summarizing individual study estimates and overall estimate | We have appended them in the main text. Additional graphs were presented as supplementary material to fully describe the results. |
| √ | Table giving descriptive information for each study included | We have presented descriptive information for each study in the supplementary material. |
| √ | Results of sensitivity testing | Subgroup analyses were conducted to analyse differences between used task in each neurocognitive domain. |
| √ | Indication of statistical uncertainty of findings | We reported mean estimates for the main outcome and 95% CI. |
| **Reporting of discussion should include** | |  |
| √ | Quantitative assessment of bias | Publication biases were assessed by funnel plots visual inspections and Egger test (3). The trim and fill methods were used as sensitivity analyses to correct biases if detected. |
| √ | Justification for exclusion | Exclusion criteria and justification are described in the manuscript. |
| √ | Assessment of quality of included studies | We adapted the Newcastle-Ottawa Scale for the evaluation of cross-sectional and cohort studies. |
| **Reporting of conclusions should include** | |  |
| √ | Consideration of alternative explanations for observed results | We discussed other explanations for our findings in the discussion section. |
| √ | Generalization of the conclusions | We have addressed the generalization of the conclusions in the discussion section. |
| √ | Guidelines for future research | We have suggested possible streams of future development and research in the discussion. |
| √ | Disclosure of funding source | Funding source described at the end of the manuscript. No separate funding was necessary for the undertaking of this meta-analysis. |

# e. Methods 1. Search terms.

We performed a multi-step literature search using the following keywords: "cognit*" OR "neurocognit*" OR "social cognit*" AND "psychosis risk" OR "prodrom*" OR "ultra-high risk" OR "clinical high risk" OR "genetic high risk" OR "at risk mental state" OR "at-risk mental state" OR "basic symptoms" OR “ultra-high risk” from inception until 1st July 2020.

# **eTable 3**. Neurocognitive domains considered in the current meta-analysis (7 MATRICS domains and 8 CHR-P domains. Adapted from Fusar-Poli et al. 2012 (4) and Hauser et al. 2017(5). CHR-P, Clinical High Risk for Psychosis.

As a note, the MATRICS was not designed to be a comprehensive battery for the assessment of cognition but rather was developed with a narrower purpose of creating a brief, highly portable and tolerable consensus battery of neurocognitive domains most likely to be sensitive to both the most common (or best characterized) cognitive impairments in schizophrenia spectrum disorders and to change following targeted treatment (pharmacological or psychological). In fact, the range of cognitive impairment in schizophrenia spectrum disorders is broader and more complex than indexed by the MATRICS, and we should expect no less of its putative clinical high-risk states. As such, in an effort to produce a comprehensive review of the extant literature, we endeavoured to be more inclusive in our approach and thus included additional neurocognitive domains and tasks that have been employed in cognitive studies of CHR-P to date and which are frequently assessed in clinical settings. While the MATRICS represents an organizing framework, it is necessarily limited such that any comprehensive meta-analysis requires the inclusion of a broader set of neurocognitive domains and tests that adhere to a related but non-redundant categorizing scheme.

| **Neurocognitive domains** | **Tasks** |
| --- | --- |
| ***MATRICS domains*** | |
| **Processing Speed** | - Trail Making Test-Part A (TMT-A)(6) - Brief Assessment of Cognition Scale Symbol Coding (BACS SC)(7) - Animal Fluency(8) - Letter Fluency(9) - Digit symbol coding test (DST)(10) - Stroop Test: Word (Stroop W)(11) - Stroop Test: Colour (Stroop C)(11) |
| **Attention/Vigilance** | - Continuous Performance Test – Identical Pairs (CPT-IP)*(12, 13) |
| **Working Memory** | - Wechsler Memory Scale-III Spatial Span Subtest (WMS-III: SS)(14) - Letter Number Span (LNS)(15) - Letter Number Sequencing Test (LNST)(10) - Arithmetic (any WAIS)(14, 16) - Self-Ordered Pointing Task (SOPT)(17) |
| **Verbal Learning** | - Hopkins Verbal Learning Test—Revised (HVLT-R)**(18) - Rey Auditory Verbal Learning Test (RAVLT)***(19) - California Verbal Learning Test I/II (CVLT)****(19-21) |
| **Visual learning** | - Brief Visuospatial Memory Test-Revised (BVMT-R)**(22) - Wechsler Memory Scale Immediate Visual Memory (WMS VM)(14) - Rey–Osterrieth Complex Figure test Immediate Recall (ROCF)(23) |
| **Reasoning and Problem-Solving** | - Neuropsychological Assessment Battery Mazes (NAB Mazes)(24) |
| **Social cognition+** | - Reading the Mind in the Eyes Test (RMET) (25, 26) - Degraded Facial Affect Recognition (DFAR)(27) - Hinting (28) |
| ***CHR-P domains*** | |
| **General intelligence IQ** | - Wechsler Adult Intelligence Scale- 3^rd^ edition (WAIS-III)(14) - Wechsler Adult Intelligence Scale-Revised (WAIS-R)(29) - Wechsler Intelligence Scale for Children- 3^rd^ edition (WISC-III) (30) |
| **Premorbid IQ** | - National Adult Reading Test (NART)(31) - MehrfachWortschaftz-Intelligenz Test-part B (MWT-B)(32) |
| **Visuospatial ability** | - WAIS/WISC Block Design (WAIS/WISC BD)(10, 30) |
| **Verbal memory** | - RAVLT Delayed Recall (RAVLT DR)(19) |
| **Visual memory** | - ROCF Delayed Recall (ROCF DR)(23) - Wechsler Memory Scale Visual Reproduction Delayed Recall (WMS VR)(14) |
| **Executive functioning** | - Trail Making Test- Part B (TMT-B)(6) - Wisconsin Card Sorting Test (WCST)(33): categories, number of correct responses, perseverative errors and perseverative responses - Stroop Test: Interference(34) |
| **Motor functioning** | - Finger Tapping Test (Tapping)(35) |
| **Olfaction** | - University of Pennsylvania Smell Identification Test (UPSIT)(36) |

**^+^**Social cognition encompassed: (a) emotional processing, (b) social perception and knowledge, (c) theory of mind, and (d) attributional bias). *****Mean d' across conditions; **Total Learning Trials 1-3; ***Learning Trials; **** Trials 1-5 Total Correct

Only tasks with 3 or more available studies in the dataset are listed

# **eTable 4**. Risk of bias (quality) assessment using modified Newcastle-Ottawa Scale for cross-sectional and cohort studies

| **Newcastle-Ottawa Scale Criteria** | **Maximum Score** |
| --- | --- |
| *Cross-Sectional Studies* | |
| Sample representative of target sample (e.g. all eligible or random sample)? | 2 |
| Sample size justified and satisfactory? | 1 |
| Non-response rate is defined satisfactory. and characteristics of responders/non-responders compared? | 1 |
| Ascertainment of exposure (i.e. menstrual cycle) is valid and/or well described? | 1 |
| Assessment of outcome with robust tool and/or record linkage? | 2 |
| Outcome per group reported appropriately? | 1 |
| *Cohort Studies* | |
| Representativeness of exposed cohort (e.g. total population or random sample. selected group) | 1 |
| Method used to ascertain exposure (menstrual cycle phase) is robust? | 1 |
| Exposed and unexposed are matched or adjustment for confounding factors? | 2 |
| Assessment of outcome was blind to exposure status or used record linkage. were robust tools used? | 2 |
| Follow-up period was sufficiently long for outcomes to occur (e.g. more than one menstrual cycle? | 1 |
| Loss to follow-up rate is reported. low (<30%). and same in exposed and non-exposed? | 1 |

# **eTable 5**. Characteristics of included studies. CHR-P, Clinical High Risk for Psychosis; HC, Healthy Controls; FEP, First Episode Psychosis; FUP, Follow-up; mo, months; NOS, Newcastle-Ottawa Scale.

| **Author year** | **Country** | **N CHR-P** | **N HC** | **N FEP** | **Age mean (SD)** | **FUP (mo)** | **NOS** | **Task used** |
| --- | --- | --- | --- | --- | --- | --- | --- | --- |
| Addington 2012(37) | Canada-USA | 146 | 85 |  | CHR-P 19.8 (4.5)  HC 19.4 (3.5) | 24 | 6 | WAIS-III/WISC-III |
| Addington 2017(38) | Canada-USA | 145 |  |  | CHR-P 19.84 (4.7) | 172 | 6 | Animal Fluency, DST, TMT-A. LNST, WAIS-III/WISC-III, WCST |
| Atkinson 2017(39) | Austria | 102 | 62 |  | CHR-P 18.6 (2.7)  HC 19.1 (3.2) | 0 | 6 | LNST, CVLT-II, UPSIT, RMET, Hinting |
| Becker 2010(40) | The Netherlands | 41 | 17 |  | CHR-P 19.85 (3.6)  HC 19.4 (3.8) | 18 | 6 | CPT-IP, NART, ROCF, Tapping |
| Blanchard 2010(41) | Ireland | 17 | 20 |  | CHR-P 12 (6.9)  HC 12.58 (4.2) | 0 | 4 | TMT-A, TMT- B |
| Bolt 2019(42) | Australia | 294 |  |  | CHR-P 19.13 (4.5) | 41 | 7 | WAIS-III |
| Brewer 2003(43) | Australia | 81 | 31 |  | CHR-P 20.26 (3.5)  HC 21.1 (3.9) | 18 | 7 | NART, UPSIT |
| Brockhous-Dumke 2005(44) | Germany | 43 | 33 |  | CHR-P 25.4 (5.8)  HC 24.5 (3.3) | 0 | 4 | WCST |
| Broome 2012(45) | UK | 28 |  |  | CHR-P 24.41 (4.2) | 21 | 6 | NART |
| Chu 2019(46) | Hong Kong | 71 | 68 | 69 | CHR-P 20.8 (6.5)  HC 24.5 (8.0)  FEP 23.87 (7.2) | 0 | 6 | LNS, WMS VR |
| Chung 2008(47) | South Korea | 33 | 36 |  | CHR-P 20.88 (3.2)  HC 21.97 (2.5) | 0 | 8 | WCST |
| Corcoran 2015(48) | USA | 49 | 31 |  | CHR-P 20.6 (3.8)  HC 21.4 (3.1) | 0 | 7 | WAIS-III |
| Couture 2008(49) | Canada-USA | 88 | 41 |  | CHR-P 18.9 (4.6)  HC 24.9 (5.1) | 0 | 7 | RMET |
| Cui 2020(50) | China. | 217 | 133 |  | CHR-P 18.56 (4.9)  HC 18.77 (4.3) | 12 | 8 | BACS SC, Animal Fluency, TMT-A, CPT-IP, WMS-III: SS, HVLT-R, BVMT-R, NAB mazes |
| Eastvold 2007(51) | USA | 40 | 36 | 15 | CHR-P 20.8 (3.5)  HC 21.8 (3.4)  FEP 21.5 (5.2) |  | 8 | WCST, Stroop W, Stroop C, Stroop interference |
| Egloff 2019(52) | Switzerland | 59 |  | 31 | CHR-P 25 (6.0)  FEP 27.3 (6.0) | 0 | 7 | CVLT-I |
| Eisenacher 2018(53) | Germany | 38 | 38 |  | CHR-P 22.9 (4.2)  HC 24.3 (5.8) | 0 | 6 | HVLT-R, BVMT-R, MWT-B, TMT- B, WCST, NAB Mazes |
| Epstein 2014(54) | USA | 21 | 55 |  | CHR-P 16.1 (3.3)  HC 16.5 (2.6) | 0 | 6 | CPT-IP |
| Frommann 2011(55) | Germany | 89 | 87 |  | CHR-P 25.3 (6.4)  HC 25.5 (4.4) | 0 | 7 | DS, Letter Fluency, TMT-A, CPT-IP, LNST, RAVLT, MWT-B, SOPT, RAVLT, TMT- B |
| Gill 2013(56) | USA | 71 | 36 |  | CHR-P 19.33 (3.6)  HC 21.7 (4.6) | 0 | 6 | WAIS-III, UPSIT |
| Goghari 2014(57) | UK | 96 | 23 | 28 | CHR-P 23.3 (4.4)  HC 25.2 (5.4)  FEP 22.6 (4.4) | 0 | 5 | WAIS-III |
| Gupta 2014(58) | USA | 53 | 35 |  | CHR-P 18.6 (2.1)  HC 18.8 (1.7) | 0 | 7 | BACS SC, TMT-A, BVMT-R, WMS-III: SS |
| He 2019(59) | China | 190 | 37 |  | CHR-P 20.47 (4.6)  HC 22.08 (3.35) | 0 | 6 | TMT-A, TMT- B |
| Healey 2013(60) | USA | 147 | 85 |  | CHR-P 19.79 (4.7)  H19.41 (4.08) | 24 | 6 | RMET |
| Hou 2016(61) | China-Australia | 40 | 40 | 40 | CHR-P 29.1 (7.0)  HC 24.4 (5.1)  FEP 26.4 (6.5) | 0 | 7 | DST, Stroop C, Stroop W, TMT-A, HVLT-R |
| Hur 2012(62) | South Korea | 41 | 40 |  | CHR-P 20.95 (3.8)  HC 21.2 (3.1) | 0 | 6 | Stroop interference |
| Hur 2013(63) | South Korea | 55 | 58 |  | CHR-P 21.96 (3.3)  HC 23.1 (2.96) | 0 | 6 | WCST |
| Hwang 2019(64) | South Korea | 40 | 85 | 85 | CHR-P 20.55 (3.0)  HC 21.24 (2.3)  FEP 21.87 (3.6) | 0 | 6 | WCST |
| Ilonen 2010(65) | Finland | 22 | 187 |  | CHR-P 15.7 (1.7)  H 15.5 (1.7) | 0 | 5 | WAIS-R/WISC-III |
| Jahshan 2010(66) | USA | 46 | 29 | 18 | CHR-P 19.8 (4.1)  HC 19.9 (5.7)  FEP 21 (5.7) | 6-36 | 5 | Stroop W, Stroop C, Stroop interference |
| Kamath 2014(67) | USA | 10 | 17 |  | CHR-P 19.9 (2.6)  H 21 (2.5) | 0 | 5 | UPSIT |
| Kang 2018(68) | South Korea | 65 | 83 |  | CHR-P 20.1 (3.4)  HC 20.8 (3.6) | 0 | 6 | WAIS-III (Korean Version) |
| Kim 2011(69) | South Korea | 45 | 49 |  | CHR-P 21.07 (3.9)  HC 22.7 (3.5) | 0 | 7 | Stroop C, TMT-A. CVLT-I, WMS-III: Spatial Span |
| Kim 2019(70) | South Korea | 60 | 71 | 47 | CHR-P 20.3 (3.5)  HC 22 (3.4)  FEP 23 (4.1) | 0 | 7 | TMT-A, CVLT-I, ROCF, TMT- B, WCST |
| Koren 2019(71) | Israel | 21 | 34 |  | CHR-P 15.9 (1.4)  HC 15.8 (1.0) | 0 | 6 | RAVLT, WAIS-R, WCST, Hinting |
| Korver 2010(72) | The Netherlands | 29 | 30 |  | CHR-P 18.8 (2.4)  HC 19.8 (3.4) | 0 | 6 | Letter Fluency, CVLT-I |
| Koshiyama 2018(73) | Japan | 30 | 20 | 26 | CHR-P 20.8 (4.0)  HC 23 (5.0)  FEP 23.9 (6.4) | 0 | 5 | NART |
| Koutsoluleris 2011(74) | Germany | 48 | 30 |  | CHR-P 24.7 (5.8)  HC 26 (2.7) | 0 | 6 | DST, Letter Fluency, TMT-A, LNS, LNST, RAVLT, MWT-B, SOPT, RAVLT, TMT- B |
| Kristensen 2019(75) | Denmark | 116 | 49 |  | CHR-P 23.8 (4.2)  HC 24.4 (3.4) | 0 | 7 | BACS SC, WAIS-III |
| Lee 2014(76) | South Korea | 75 | 75 |  | CHR-P 19.97 (3.8)  HC 19.9 (2.5) | 0 | 7 | TMT-A, ROCF, K-WAI, TMT- B, WCST |
| Lee 2015(77) | South Korea | 40 | 46 | 24 | CHR-P 19.9 (3.6)  HC 20.8 (3.5)  FEP19.9 (3.6) | 0 | 6 | WAIS-III (Korean Version) |
| Lepock 2019(78) | Canada | 36 | 21 |  | CHR-P 21.7 (3.0)  HC 21.3 (3.4) | 0 | 5 | NART |
| Li 2018(79) | China. | 34 | 37 |  | CHR-P 21.5 (3.5)  HC 20.8 (3.1) | 0 | 7 | BACS SC, Stroop W, Stroop C, CPT-IP, HVLT-R, BVMT-R |
| Lin 2013(80) | Australia. UK | 124 | 36 |  | CHR-P 19.13 (3.3)  HC 20.75 (4.4) | 0 | 7 | DST, TMT-A, Arithmetic, RAVLT, WMS VR, WAIS-R- WAIS/WISC BD, TMT- B |
| Lindgren 2010(81) | Finland | 62 | 72 |  | CHR-P 16.5 (0.9)  HC 16.5 (0.9) | 0 | 5 | BACS SC, Animal Fluency, TMT-A, WMS-III: SS, CVLT-I, WMS VR, TMT- B, Tapping |
| Liu 2019(82) | China-USA | 73 | 72 |  | CHR-P 23.3 (4.5)  HC 24 (2.9) | 0 | 7 | WMS VM |
| Magaud 2014(83) | France | 104 | 64 | 30 | CHR-P 20.9 (3.5)  H21.3 (3.8)  FEP 22.5 (4.3) | 0 | 6 | Arithmetic, WAIS-R |
| Menghini – Muller 2019(84) | Switzerland | 343 | 67 |  | CHR-P 22.4 (4.9)  HC 22.9 (4.09) | 0 | 6 | DST, TMT-A, Arithmetic, RAVLT, WAIS-III, WAIS/WISC BD. RAVLT, TMT- B |
| Metzler 2015(85) | Switzerland | 72 |  | 12 | CHR-P 20.52 (5.9)  FEP 19.1 (4.8) | 18 | 6 | Animal Fluency, DST |
| Millman 2014(86) | USA | 37 | 35 |  | CHR-P 18.6 (1.8)  HC 18.1 (2.3) | 0 | 7 | BACS SC, Animal Fluency, TMT-A |
| Mirzakhanian 2013(87) | USA | 109 | 102 | 90 | CHR-P 19.1 (4.1)  HC 20.8 (4.6)  FEP 20.9 (5.4) | 0 | 7 | CPT-IP |
| Mittal 2010(88) | USA | 90 |  |  | CHR-P 15.64 (3.0) | 24 | 6 | WAIS-III/WISC-III |
| Modinos 2015(89) | UK | 18 | 18 | 22 | CHR-P 24.4 (4.1)  HC 27.9 (5.0)  FEP 23.8 (4.6) | 0 | 6 | NART |
| Montalvo 2014(90) | Spain | 23 | 29 | 55 | CHR-P 22.5 (4.3)  HC 26.4 (4.3)  FEP 24.5 (5.3) | 0 | 7 | HVLT-R, BVMT-R, NAB mazes |
| Ohmuro 2016(91) | Japan | 36 | 25 | 40 | CHR-P 20.9 (4.7)  HC 21.3 (1.0)  FEP 22.9 (6.3) | 0 | 7 | NART |
| Ohmuro 2018(92) | Japan | 50 | 29 |  | CHR-P 20 (4.1)  HC 21.2 (1.0)  FEP 22.9 (6.3) | 0 | 7 | NART, WCST |
| Pflueger 2018(93) | Switzerland | 116 | 57 | 90 | CHR-P 25.7 (6.5)  HC 24.9 (6.4)  FEP 28.3 (7.9) | 0 | 5 | CVLT-I, WCST |
| Pukrop 2006(94) | Germany | 128 | 179 |  | CHR-P 24.43 (6.0)  HC 29.23 (8.4) | 0 | 5 | MWT-B |
| Pukrop 2007(95) | Germany | 39 | 44 |  | CHR-P 24.92 (5.3)  HC 25.08 (3.2) | 0 | 5 | DST, CPT-IP, LNS, LNST, SOPT, ROCF, TMT- B, WCST |
| Randers 2020(96) | Denmark | 220 | 50 |  | CHR-P 22.4 (3.3)  HC 23.5 (4.4) | 0 | 5 | TMT-A, WMS-III: SS, WAIS/WISC BD, TMT- B, DFAR |
| Sanada 2018(97) | Spain-Japan | 13 | 30 |  | CHR-P 22.2 (8.0)  HC 24 (6.3) | 6 | 7 | Stroop W, Stroop C, Stroop Interference, LNST, WCST |
| Seidman 2010(98) | USA-Canada | 167 | 109 |  | CHR-P 18.31 (4.73)  HC 18.8 (4.5) | 30 | 6 | CPT-IP, WAIS-R, TMT- B, WCST |
| Seidman 2016(99) | USA-Canada | 689 | 264 |  | CHR-P 18.5 (4.2)  HC 19.8 (4.7) | 24 | 5 | BACS SC, Animal Fluency, CPT-IP, WMS-III: Spatial Span, LNS, HVLT-R, BVMT-R, WAIS/WISC BD, UPSIT, NAB Mazes |
| Shin 2016(100) | South Korea | 47 | 28 |  | CHR-P 19.3 (3.3)  HC 27 (6.0) | 24 | 5 | Letter Fluency |
| Simon 2007(101) | Switzerland | 93 | 49 | 43 | CHR-P 20.81 (5.0)  HC 21.8 (4.9) | 0 | 5 | Animal Fluency, Letter Fluency, LNS, RAVL, MWT-B, RAVLT, TMT- B, WCST |
| Standford 2011(102) | USA | 63 | 38 |  | CHR-P 19.6 (3.6)  HC 23.98 (7.5) | 0 | 5 | WAIS-R, RMET |
| Studerus 2018(103) | Switzerland | 168 | 109 |  | CHR-P 25.4 (7.2)  HC 25 (5.3) | 0 | 5 | CVLT |
| Szily 2009(104) | Hungary | 26 | 50 |  | CHR-P 22 (8.7)  HC 21.1 (6.3) | 0 | 5 | WAIS-R, RMET |
| Takahashi 2018(105) | Japan | 38 | 61 |  | CHR-P 18.4 (3.9)  HC 25.6 (3.2) | 0 | 5 | NART |
| Thompson 2012(106) | Australia | 30 | 30 | 40 | CHR-P 19.1 (2.8)  HC 19.3 (2.9) | 0 | 6 | WMS-III: Spatial Span, LNST, WAIS-III, Hinting |
| Tognin 2020(107) | UK | 309 | 51 |  | CHR-P 22.63 (4.8)  HC 23.37 (3.9) | 24 | 7 | WAIS-III, DFAR |
| Tor 2019(108) | Spain | 81 | 39 |  | CHR-P 15.11 (1.8)  HC 15.58 (1.5) | 0 | 7 | TMT-A, WMS VM, ROCF, WAIS-III, TMT- B |
| Üçok 2013(109) | Tukey | 81 | 35 | 53 | CHR-P 22.13 (6.0)  HC 20 (3.7) | 0 | 5 | TMT-A, TMT- B, WCST, Stroop C, Stroop interference |
| Van Rijin 2011(110) | The Netherlands | 36 | 21 |  | CHR-P 15.2 (2.1)  HC 15.9 (1.4) | 0 | 5 | DFAR |
| Wood 2007(111) | Australia | 16 | 17 |  | CHR-P 19.4 (3.5)  HC 19.7 (2.4) | 12 | 5 | RAVLT, WMS VM |
| Woodberry 2013(112) | USA | 53 | 32 |  | CHR-P 16.3 (2.6)  HC 16 (2.4) | 12 | 6 | CPT-IP, CVLT-I, WCST, Tapping |
| Zhang 2016(113) | China. | 83 | 90 |  | CHR-P 20.3 (1.7)  HC 19.1 (2.0) | 12 | 5 | RMET |
| Ziermans 2014(114) | The Netherlands | 43 | 44 |  | CHR-P 15.22 (2.2)  HC 15.4 (1.3) | 72 | 5 | Letter Fluency, CPT-IP, WAIS-III, WCST, Tapping |

CHR-P Clinical High risk for Psychosis; HC healthy controls; FEP First Episode Psychosis

TMT-A Trail Making Test-Part A; BACS SC Basic Assessment of Cognition Scale Symbol Coding; DST Digit Symbol coding test; CPT-IP Continuous Performance Test Identical Pairs; WMS-III: SS Wechsler Memory Scale. 3^rd^ ed. spatial span subtest; LNS Letter Number Span; LNST Letter Number Sequencing Test; HVLT-R Hopkins Verbal Learning Test-Revised; RAVLT Rey Auditory Verbal Learning Test; CVLT California Verbal Learning Test; BVMT-R Brief Visuospatial Memory Test-Revised; ROCT Rey- Osterrieth Complex Fiure Test; NAB Mazes Neuropsychological Assessment Battery Mazes; DFAR Degraded Facial Affect Recognition; RMET Reading the Mind in the Eyes; WAIS Wechsler Intelligence Scale; NART National Adult Reading Test; MWT-B. MehrfachWortschaftz-Intelligenz Test-part B; SOPT Self-Ordered Pointing Task; RAVLT DR Rey Auditory Verbal Learning Test Delayed Recall; ROCF Rey- Osterrieth Complex Figure Test; WMS VR Weschler Memory Scale Visual Reproduction Delayed Recall; TMT-B Trail Making Test-Part B; WCST categories Wisconsin Card Sorting Test categories; WCST number of corrects responses Wisconsin Card Sorting Test number of correct responses; WCST perseverative errors Wisconsin Card Sorting Test perseverative errors; WCST perseverative responses Wisconsin Card Sorting Test perseverative responses; UPSIT University of Pennsylvania Smell Identification Test

# **eTable 6**. Coefficient of Variation Rations (CVR) analyses.

| ***CHR-P vs HC*** |  |  |  |  |  |  |
| --- | --- | --- | --- | --- | --- | --- |
| ***Task*** | ***CVR*** | ***95% CI []*** | ***se*** | ***z*** | ***p*** | ***Q p*** |
| *Animal fluency* | 1.09 | [0.93, 1.27] | 0.0793 | 10.953 | 0.2734 | 9.9915. p-val = 0.0406 |
| *BACS SC* | 1.21 | [0.96, 1.52] | 0.1177 | 16.190 | 0.1054 | 14.9839. p-val = 0.0047 |
| *DST* | 1.14 | [1.03, 1.27] | 0.0538 | 24.964 | **0.0125** | 6.6347. p-val = 0.2493 |
| *Letter fluency* | 1.12 | [0.93, 1.34] | 0.0941 | 11.805 | 0.2378 | 9.8756. p-val = 0.0788 |
| *Stroop C* | 1.38 | [1.03, 1.85] | 0.1500 | 21.478 | **0.0317** | 12.8918. p-val = 0.0118 |
| *Stroop W* | 1.71 | [1.23, 2.37] | 0.1675 | 32.019 | **0.0014** | 4.2753. p-val = 0.1179 |
| *TMT-A* | 1.31 | [1.03, 1.66] | 0.1208 | 22.334 | **0.0255** | 96.1030. p-val < .0001 |
| *CPT-IP* | 1.19 | [1.06, 1.32] | 0.0551 | 30.921 | **0.002** | 21.8203. p-val = 0.0160 |
| *Arithmetic* | 1.14 | [0.99, 1.30] | 0.0695 | 18.691 | 0.0616 | 0.5753. p-val = 0.7500 |
| *LNS* | 1.18 | [1.09, 1.29] | 0.0434 | 38.398 | **0.0001** | 3.7309. p-val = 0.4436 |
| *LNST* | 1.15 | [1.01, 1.31] | 0.0676 | 20.670 | **0.0387** | 4.2099. p-val = 0.3783 |
| *SOPT* | 0.97 | [0.77, 1.21] | 0.1135 | -0.2906 | 0.7714 | 1.4846. p-val = 0.4760 |
| *WMS-III: SS* | 1.19 | [1.10, 1.29] | 0.0402 | 44.028 | **<.0001** | 4.7156. p-val = 0.3177 |
| *CVLT* | 1.6 | [1.35, 1.91] | 0.0895 | 52.741 | **<.0001** | 16.1407. p-val = 0.0130 |
| *RAVLT* | 1.41 | [1.09, 1.82] | 0.1318 | 25.895 | **0.0096** | 19.6511. p-val = 0.0015 |
| *HVLT-R* | 1.36 | [1.14, 1.61] | 0.0882 | 34.775 | **0.0005** | 16.6426. p-val = 0.0107 |
| *BVMT-R* | 1.52 | [1.28, 1.80] | 0.0885 | 47.098 | **<.0001** | 23.9994. p-val = 0.0002 |
| *WMS VM* | 1.18 | [1.05, 1.32] | 0.0585 | 27.644 | **0.0057** | 4.1234. p-val = 0.3896 |
| *ROCF IR* | 1.28 | [0.78, 2.10] | 0.2508 | 0.9911 | 0.3216 | 19.7223. p-val < .0001 |
| *NAB Mazes* | 1.33 | [1.09, 1.62] | 0.0996 | 28.582 | **0.0043** | 12.9016. p-val = 0.0049 |
| *DFAR neutral* | 1.00 | [1.61, 1.65] | 0.2553 | -0.0018 | 0.9986 | 11.6881. p-val = 0.0029 |
| *DFAR happy* | 0.5 | [0.14, 1.81] | 0.6513 | -1.053 | 0.2925 | 124.7725. p-val < .0001 |
| *DFAR anger* | 0.46 | [0.09, 2.30] | 0.8180 | -0.9391 | 0.3477 | 176.7324. p-val < .0001 |
| *DFAR fear* | 0.47 | [0.09, 2.35] | 0.8193 | -0.9195 | 0.3578 | 71.9755. p-val < .0001 |
| *RMET* | 1.06 | [0.94, 1.20] | 0.0627 | 0.9326 | 0.3510 | 1.9892. p-val = 0.5746 |
| *Hinting* | 1.74 | [1.39, 2.18] | 0.1149 | 48.223 | **<.0001** | 2.9152. p-val = 0.2328 |
|  |  |  |  |  |  |  |
| *IQ* | 0.87 | [0.55, 1.39] | 0.2382 | -0.5715 | 0.5677 | 853.4277. p-val < .0001 |
| *IQ verbal* | 1.2 | [0.92, 1.58] | 0.1377 | 13.457 | 0.1784 | 5.7643. p-val = 0.0560 |
| *IQ performance* | 1.41 | [1.12, 1.78] | 0.1191 | 28.761 | **0.0040** | 4.4117. p-val = 0.1102 |
| *MWT-B* | 1.25 | [0.92, 1.7] | 0.1572 | 14.273 | 0.1535 | 33.9456. p-val < .0001 |
| *NART* | 1.4 | [1.13, 1.73] | 0.1073 | 31.257 | **0.0018** | 13.7564. p-val = 0.0325 |
| *WAIS/WISC BD* | 1.21 | [1.11, 1.31] | 0.0440 | 42.633 | **<.0001** | 3.6289. p-val = 0.4585 |
| *RAVLT DR* | 1.52 | [0.9, 2.57] | 0.2686 | 15.567 | 0.1196 | 42.2161. p-val < .0001 |
| *ROCF DR* | 1.29 | [0.9, 1.85] | 0.1842 | 13.861 | 0.1657 | 14.2622. p-val = 0.0026 |
| *WMS VR* | 2.55 | [1.06, 6.14] | 0.4484 | 20.880 | **0.0368** | 33.2866. p-val < .0001 |
| *TMT- B* | 1.17 | [1.00, 1.37] | 0.0814 | 19.104 | 0.0561 | 70.3405. p-val < .0001 |
| *WCST Perseverative Responses* | 0.98 | [0.78, 1.27] | 0.1250 | -0.0531 | 0.9576 | 4.1704. p-val = 0.2436 |
| *WCST categories* | 1.28 | [1.06, 1.54] | 0.0967 | 25.339 | **0.0113** | p-val = 0.0458 |
| *WCST perseverative errors* | 1.01 | [0.91, 1.13] | 0.0533 | 0.2521 | 0.8010 | p-val = 0.9686 |
| *Stroop interference* | 1.11 | [0.72, 1.69] | 0.2166 | 0.4734 | 0.6359 | 23.5055. p-val = 0.0001 |
| *WCST number of correct responses* | 1.53 | [1.03, 2.26] | 0.2000 | 21.156 | 0.0344 | 13.2597. p-val = 0.0041 |
| *Tapping* | 1.09 | [0.93, 1.27] | 0.0783 | 10.633 | 0.2876 | 2.5759. p-val = 0.4617 |
| *UPSIT* | 1.05 | [0.88, 1.26] | 0.0901 | 0.5604 | 0.5752 | 8.2553. p-val = 0.0827 |
| ***CHR-P transitioned vs non transitioned*** | | | | | | |
| *Animal fluency* | 1.19 | [1.01, 1.4] | 0.0841 | 20.287 | **0.0425** | 4.3549. p-val = 0.2256 |
| *CPT-IP* | 1.13 | [0.87, 1.47] | 0.1348 | 0.9063 | 0.3648 | 12.6841. p-val = 0.0129 |
| *CVLT* | 1.43 | [0.99, 2.06] | 0.1866 | 19.238 | 0.0544 | 0.9172. p-val = 0.6322 |
| *DST* | 1.05 | [0.90, 1.22] | 0.0793 | 0.5669 | 0.5708 | 12.0672. p-val = 0.0605 |
| *IQ* | 1.01 | [0.88, 1.15] | 0.0682 | 0.1229 | 0.9022 | 8.6031. p-val = 0.2824 |
| *ROCF DR* | 0.89 | [0.65, 1.22] | 0.1583 | -0.7175 | 0.4731 | 3.5626. p-val = 0.1684 |
| *TMT-A* | 0.97 | [0.82, 1.13] | 0.0816 | -0.431 | 0.6665 | 2.3850. p-val = 0.6653 |
| *Tapping* | 1.03 | [0.77, 1.37] | 0.1471 | 0.1753 | 0.8608 | 0.9229. p-val = 0.6304 |
| *LNST* | 0.98 | [0.62, 1.55] | 0.2355 | -0.1022 | 0.9186 | 21.3944. p-val = 0.0003 |
| *UPSIT* | 1.19 | [0.83, 1.72] | 0.1869 | 0.9407 | 0.3468 | 9.3201. p-val = 0.0253 |
| *WCST perseverative errors* | 1.21 | [1.01, 1.46] | 0.0956 | 20.241 | **0.0430** | 3.4387. p-val = 0.4873 |
| *NART* | 1.49 | [0.74, 2.99] | 0.3558 | 11.172 | 0.2639 | 13.1472. p-val = 0.0014 |
| ***CHR-P vs FEP*** |  |  |  |  |  |  |
| *TMT-A* | 0.86 | [0.57, 1.30] | 0.2095 | -0.701 | 0.4832 | 9.5689. p-val = 0.0084 |
| *HVLT* | 1.2 | [0.97, 1.48] | 0.1074 | 16.868 | 0.0916 | 1.0687. p-val = 0.5861 |
| *CVLT* | 1.24 | [1.07, 1.44] | 0.0749 | 29.018 | **0.0037** | 0.3243. p-val = 0.8503 |
| *IQ* | 1.12 | [0.90, 1.39] | 0.1096 | 10.275 | 0.3042 | 2.7996. p-val = 0.2467 |
| *NART* | 0.79 | [0.56, 1.11] | 0.1768 | -1.3469 | 0.1780 | 4.7854. p-val = 0.0914 |
| *TMT- B* | 0.91 | [0.75, 1.09] | 0.0953 | -1.012 | 0.3116 | 1.6518. p-val = 0.4378 |
| *WCST Categories* | 1.81 | [1.18, 2.79] | 0.2191 | 27.207 | **0.0065** | 9.9987. p-val = 0.0067 |
| *WCST Perseverative Errors* | 1.05 | [0.88, 1.25] | 0.0895 | 0.5272 | 0.5981 | 3.0044. p-val = 0.3909 |
| *Stroop interference* | 1.1 | [0.90, 1.35] | 0.1045 | 0.9429 | 0.3458 | 0.6160. p-val = 0.7349 |

TMT-A Trail Making Test-Part A; BACS SC Basic Assessment of Cognition Scale Symbol Coding; DST Digit Symbol coding test; CPT-IP Continuous Performance Test Identical Pairs; WMS-III: SS Wechsler Memory Scale. 3^rd^ ed. spatial span subtest; LNS Letter Number Span; LNST Letter Number Sequencing Test; HVLT-R Hopkins Verbal Learning Test-Revised; RAVLT Rey Auditory Verbal Learning Test; CVLT California Verbal Learning Test; BVMT-R Brief Visuospatial Memory Test-Revised; ROCT Rey- Osterrieth Complex Fiure Test; NAB Mazes Neuropsychological Assessment Battery Mazes; DFAR Degraded Facial Affect Recognition; RMET Reading the Mind in the Eyes; WAIS Wechsler Intelligence Scale; NART National Adult Reading Test; MWT-B. MehrfachWortschaftz-Intelligenz Test-part B; SOPT Self-Ordered Pointing Task; RAVLT DR Rey Auditory Verbal Learning Test Delayed Recall; ROCF Rey- Osterrieth Complex Figure Test; WMS VR Weschler Memory Scale Visual Reproduction Delayed Recall; TMT-B Trail Making Test-Part B; WCST categories Wisconsin Card Sorting Test categories; WCST number of corrects responses Wisconsin Card Sorting Test number of correct responses; WCST perseverative errors Wisconsin Card Sorting Test perseverative errors; WCST perseverative responses Wisconsin Card Sorting Test perseverative responses; UPSIT University of Pennsylvania Smell Identification Test

# eTable 7. Metaregressions CHR-P vs HC

|  | **k** | **ß** | **SE** | **z** | **p** | **95% CI** |
| --- | --- | --- | --- | --- | --- | --- |
| **Processing Speed** | | | | | | |
| Age | 27 | -0.0618 | 0.0229 | -2.6944 | **0.0071** | -0.1067, -0.0168 |
| Sex male | 27 | -0.0111 | 0.0085 | -1.3051 | 0.1918 | -0.0277, 0.0055 |
| Years of education | 16 | -0.0457 | 0.0445 | -1.0274 | 0.3042 | -0.1330, 0.0415 |
| Caucasian | n.a. |  |  |  |  |  |
| GAF | 12 | 0.0075 | 0.0129 | 0.5810 | 0.5613 | -0.0178, 0.0327 |
| Attenuated psychotic positive symptoms | 8 | 0.0565 | 0.0635 | 0.8900 | 0.3735 | -0.0680, 0.1810 |
| Attenuated psychotic negative symptoms | 8 | -0.0162 | 0.0389 | -0.4151 | 0.6781 | -0.0925, 0.0602 |
| NOS | 27 | -0.0418 | 0.0622 | -0.6725 | 0.5013 | -0.1637, 0.0800 |
| Baseline AP exposure | n.a. |  |  |  |  |  |
| Type of CHR-P instrument | 23 |  |  |  |  |  |
|  | CAARMS | 1.2907 | 0.1834 | 7.0393 | **<.0001** | 0.9313, 1.6500 |
|  | SIPS | 1.5090 | 0.1519 | 9.9362 | **<.0001** | 1.2113, 1.8067 |
|  | BSABS | 1.4045 | 0.5515 | 2.5466 | **0.0109** | 0.3235, 2.4855 |
|  | BSIP | 0.9954 | 0.5385 | 1.8487 | 0.0645 | -0.0599 ,2.0508 |
|  | Others | 1.5090 | 0.1519 | 9.9362 | **<.0001** | 1.2113, 1.8067 |
| Type of CHR-P | 12 |  |  |  |  |  |
|  | APS | -0.0023 | 0.0059 | -0.3924 | 0.6947 | -0.0140, 0.0093 |
|  | BLIP | -0.0010 | 0.0069 | -0.1483 | 0.8821 | -0.0145, 0.0125 |
|  | GRD | -0.0047 | 0.0066 | -0.7113 | 0.4769 | -0.0178, 0.0083 |
| **Working memory** | | | | | | |
| Age | 16 | 0.0273 | 0.0195 | 1.4012 | 0.1612 | -0.0109, 0.0655 |
| Sex male | 16 | 0.0090 | 0.0051 | 1.7843 | 0.0744 | -0.0009, 0.0189 |
| Years of education | 11 | 0.0256 | 0.0193 | 1.3265 | 0.1847 | -0.0122, 0.0635 |
| Caucasian | n.a. |  |  |  |  |  |
| GAF | n.a |  |  |  |  |  |
| Attenuated psychotic positive symptoms | n.a |  |  |  |  |  |
| Attenuated psychotic negative symptoms | n.a |  |  |  |  |  |
| NOS | 16 | -0.0056 | 0.0462 | -0.1209 | 0.9038 | -0.0961, 0.0849 |
| Baseline AP exposure | n.a |  |  |  |  |  |
| Type of CHR-P instrument | 13 |  |  |  |  |  |
|  | CAARMS | 0.0042 | 0.0900 | 0.0462 | 0.9631 | -0.1723, 0.1806 |
|  | SIPS | 0.1949 | 0.1260 | 1.5466 | 0.1220 | -0.0521, 0.4419 |
|  | Others | 0.0924 | 0.2386 | 0.3874 | 0.6985 | -0.3752, 0.5600 |
| **Verbal learning** | | | | | | |
| Age | 20 | -0.0333 | 0.0189 | -1.7599 | 0.0784 | -0.0704, 0.0038 |
| Sex male | 20 | -0.0000 | 0.0070 | -0.0011 | 0.9991 | -0.0138, 0.0138 |
| Years of education | 12 | -0.0034 | 0.0519 | -0.0650 | 0.9482 | -0.1051, 0.0983 |
| Caucasian | n.a |  |  |  |  |  |
| GAF | 8 | 0.0050 | 0.0127 | 0.3945 | 0.6932 | -0.0198, 0.0298 |
| Attenuated psychotic positive symptoms | n.a. |  |  |  |  |  |
| Attenuated psychotic negative symptoms | n.a. |  |  |  |  |  |
| NOS | 20 | -0.0548 | 0.0481 | -1.1398 | 0.2544 | -0.1491, 0.0395 |
| Baseline AP exposure | 7 | -0.0030 | 0.0039 | -0.7858 | 0.4320 | -0.0106, 0.0046 |
| Type of CHR-P instrument | 18 |  |  |  |  |  |
|  | CAARMS | -0.3036 | 0.2446 | -1.2408 | 0.2147 | -0.7831, 0.1759 |
|  | SIPS | -0.0461 | 0.2388 | -0.1931 | 0.8469 | -0.5142, 0.4219 |
|  | Others | -0.4036 | 0.2810 | -1.4363 | 0.1509 | -0.9543 ,0.1472 |
| Type of CHR-P | 7 |  |  |  |  |  |
|  | APS | -0.0052 | 0.0099 | -0.5265 | 0.5985 | -0.0246, 0.0142 |
|  | BLIP | -0.0005 | 0.0090 | -0.0605 | 0.9517 | -0.0181, 0.0170 |
|  | GRD | -0.0170 | 0.0168 | -1.0123 | 0.3114 | -0.0498, 0.0159 |
| **Visual learning** | | | | | | |
| Age | 13 | -0.0182 | 0.0215 | -0.8478 | 0.3966 | -0.0604, 0.0239 |
| Sex male | 13 | -0.0033 | 0.0056 | -0.5997 | 0.5487 | -0.0143, 0.0076 |
| Years of education | 8 | -0.1655 | 0.1133 | -1.4600 | 0.1443 | -0.3876, 0.0567 |
| Caucasian | n.a. |  |  |  |  |  |
| GAF | n.a. | -0.0164 | 0.0207 | -0.7918 | 0.4285 | -0.0570, 0.0242 |
| Attenuated psychotic positive symptoms | 8 | 0.0607 | 0.0561 | 1.0825 | 0.2790 | -0.0492, 0.1706 |
| Attenuated psychotic negative symptoms | 8 | 0.0360 | 0.0541 | 0.6651 | 0.5060 | -0.0700, 0.1420 |
| NOS | 13 | 0.0525 | 0.0512 | 1.0253 | 0.3052 | -0.0478, 0.1527 |
| Baseline AP exposure | n.a. |  |  |  |  |  |
| Type of CHR-P instrument | 12 |  |  |  |  |  |
|  | CAARMS | 0.0954 | 0.1341 | 0.7111 | 0.4770 | -0.1675, 0.3582 |
|  | SIPS | 0.1930 | 0.0709 | 2.7220 | 0.0065 | 0.0540, 0.3319 |
|  | Others | 0.2929 | 0.2409 | 1.2160 | 0.2240 | -0.1792, 0.7651 |
| **General Intelligence** | | | | | | |
| Age | 20 | -0.0359 | 0.0763 | -0.4706 | 0.6379 | -0.1855, 0.1136 |
| Sex male | 20 | -0.0037 | 0.0177 | -0.2109 | 0.8330 | -0.0385, 0.0310 |
| Years of education | 8 | 0.0723 | 0.3487 | 0.2072 | 0.8358 | -0.6112, 0.7557 |
| Caucasian | n.a. |  |  |  |  |  |
| GAF | 13 | 0.0068 | 0.0175 | 0.3910 | 0.6958 | -0.0275, 0.0411 |
| Attenuated psychotic positive symptoms | 7 | -0.0025 | 0.2379 | -0.0105 | 0.9916 | -0.4687, 0.4637 |
| Attenuated psychotic negative symptoms | 7 | -0.0788 | 0.1350 | -0.5838 | 0.5594 | -0.3433, 0.1857 |
| NOS | 20 | 0.0525 | 0.2006 | 0.2615 | 0.7937 | -0.3408, 0.4457 |
| Baseline AP exposure | 9 | 0.0034 | 0.0237 | 0.1418 | 0.8872 | -0.0430, 0.0498 |
| Type of CHR-P instrument | 19 |  |  |  |  |  |
|  | CAARMS | -0.1886 | 0.3317 | -0.5685 | 0.5697 | -0.8387, 0.4615 |
|  | SIPS | -0.1926 | 0.2533 | -0.7604 | 0.4470 | -0.6890, 0.3038 |
| Type of CHR-P | 11 |  |  |  |  |  |
|  | APS | <-0.0001 | <0.001 | -0.6061 | 0.5445 | -0.0001, 0.0001 |
|  | BLIP | -0.0001 | 0.0008 | -0.1769 | 0.8596 | -0.0017, 0.0014 |
|  | GRD | 0.0001 | 0.0002 | 0.7240 | 0.4690 | -0.0002, 0.0005 |
| **Premorbid IQ** | | | | | | |
| Age | 12 | -0.0049 | 0.0415 | -0.1185 | 0.9056 | -0.0862, 0.0764 |
| Sex male | 11 | -0.0104 | 0.0082 | -1.2766 | 0.2017 | -0.0265, 0.0056 |
| Years of education | 7 | -0.0584 | 0.1264 | -0.4616 | 0.6443 | -0.3062, 0.1894 |
| Caucasian | n.a. |  |  |  |  |  |
| GAF | 8 | 0.0035 | 0.0194 | 0.1782 | 0.8586 | -0.0346, 0.0415 |
| Attenuated psychotic positive symptoms | n.a. |  |  |  |  |  |
| Attenuated psychotic negative symptoms | n.a. |  |  |  |  |  |
| NOS | 12 | 0.0130 | 0.0614 | 0.2123 | 0.8319 | -0.1072, 0.1333 |
| Baseline AP exposure | n.a. |  |  |  |  |  |
| Type of CHR-P instrument | 12 |  |  |  |  |  |
|  | CAARMS | 0.2872 | 0.1245 | 2.3071 | **0.0210** | 0.0432, 0.5312 |
|  | SIPS | 0.3346 | 0.1014 | 3.2983 | **0.0010** | 0.1358, 0.5334 |
|  | Others | -0.0635 | 0.1619 | -0.3922 | 0.6949 | -0.3808, 0.2539 |
| **Executive functioning** |  |  |  |  |  |  |
| Age | 29 | -0.0000 | 0.0000 | -0.3184 | 0.7501 | -0.0000, 0.0000 |
| Sex male | 28 | 0.0023 | 0.0052 | 0.4465 | 0.6552 | -0.0079, 0.0125 |
| Years of education | 18 | -0.0699 | 0.0545 | -1.2815 | 0.2000 | -0.1768 ,0.0370 |
| Caucasian | n.a |  |  |  |  |  |
| GAF | 16 | -0.0068 | 0.0056 | -1.2096 | 0.2264 | -0.0179, 0.0042 |
| Attenuated psychotic positive symptoms | n.a. |  |  |  |  |  |
| Attenuated psychotic negative symptoms | n.a. |  |  |  |  |  |
| NOS | 29 | -0.0097 | 0.0435 | -0.2236 | 0.8231 | -0.0951, 0.0756 |
| Baseline AP exposure | 13 | -0.0042 | 0.0070 | -0.6041 | 0.5458 | -0.0178, 0.0094 |
| Type of CHR-P instrument | 26 |  |  |  |  |  |
|  | CAARMS | -0.1469 | 0.3282 | -0.4478 | 0.6543 | -0.7902, 0.4963 |
|  | SIPS | 0.0010 | 0.3229 | 0.0032 | 0.9974 | -0.6319, 0.6340 |
|  | BSIP | -0.3443 | 0.4244 | -0.8111 | 0.4173 | -1.1762, 0.4876 |
|  | Others | -0.1240 | 0.3759 | -0.3299 | 0.7415 | -0.8608, 0.6128 |
| Type of CHR-P |  |  |  |  |  |  |
|  | APS | 0.0045 | 0.0045 | 0.9816 | 0.3263 | -0.0044, 0.0133 |
|  | BLIP | 0.0069 | 0.0049 | 1.3995 | 0.1617 | -0.0028, 0.0166 |
|  | GRD | -0.0027 | 0.0053 | -0.5065 | 0.6125 | -0.0130, 0.0077 |

# eTable 8. Metaregressions CHR-transitioning vs CHR-non transitioning

|  | **k** | **ß** | **SE** | **z** | **p** | **95% CI** |
| --- | --- | --- | --- | --- | --- | --- |
| **Processing Speed** |  |  |  |  |  |  |
| Age | 8 | 0.0040 | 0.0305 | 0.1327 | 0.8944 | -0.0557, 0.0638 |
| Sex male | 8 | -0.0024 | 0.0086 | -0.2834 | 0.7769 | -0.0192, 0.0144 |
| Years of education | n.a |  |  |  |  |  |
| Caucasian | n. a. |  |  |  |  |  |
| GAF | n.a |  |  |  |  |  |
| Attenuated psychotic positive symptoms | n.a |  |  |  |  |  |
| Attenuated psychotic negative symptoms | n.a |  |  |  |  |  |
| NOS | 8 | 0.0322 | 0.0517 | 0.6235 | 0.5330 | -0.0691, 0.1336 |
| Baseline AP exposure | n.a |  |  |  |  |  |
| Follow-up time | 8 | -0.0001 | 0.0014 | -0.0727 | 0.9420 | -0.0028, 0.0026 |
| Type of CHR-P instrument | n.a. |  |  |  |  |  |
|  | SIPS | -0.0188 | 0.1615 | -0.1163 | 0.9074 | -0.3353, 0.2977 |
|  | SPI-A or SPI-CY |  |  |  |  |  |
|  | COPS | -0.3474 | 0.2038 | -1.7043 | 0.0883 | -0.7470, 0.0521 |
| **General intelligence** |  |  |  |  |  |  |
| Age | 8 | -0.0256 | 0.0273 | -0.9409 | 0.3468 | -0.0791, 0.0278 |
| Sex male | 8 | 0.0097 | 0.0077 | 1.2518 | 0.2106 | -0.0055, 0.0249 |
| Years of education | n. a. |  |  |  |  |  |
| Caucasian | n.a |  |  |  |  |  |
| GAF | n.a |  |  |  |  |  |
| Attenuated psychotic positive symptoms | n.a |  |  |  |  |  |
| Attenuated psychotic negative symptoms | n.a |  |  |  |  |  |
| NOS | 8 | -0.0452 | 0.1017 | -0.4447 | 0.6566 | -0.2447, 0.1542 |
| Baseline AP exposure | n.a |  |  |  |  |  |
| Follow-up time | 8 | 0.0018 | 0.0012 | 1.4804 | 0.1388 | -0.0006, 0.0042 |
| Type of CHR-P instrument | 8 |  |  |  |  |  |
|  | CAARMS | -0.1544 | 0.1591 | -0.9704 | 0.3319 | -0.4663, 0.1575 |
|  | SIPS | -0.0076 | 0.0725 | -0.1045 | 0.9167 | -0.1497, 0.1346 |

# eTable 9. Metaregressions CHR-P vs FEP

|  | **k** | **ß** | **SE** | **z** | **p** | **95% CI** |
| --- | --- | --- | --- | --- | --- | --- |
| **Executive functioning** |  |  |  |  |  |  |
| Age | 7 | -0.0472 | 0.0332 | -1.4235 | 0.1546 | -0.1122, 0.0178 |
| Sex male | 7 | 0.0123 | 0.0088 | 1.4008 | 0.1613 | -0.0049, 0.0295 |
| Years of education | n.a |  |  |  |  |  |
| Caucasian | n. a. |  |  |  |  |  |
| GAF | n. a. |  |  |  |  |  |
| Attenuated psychotic positive symptoms | n.a |  |  |  |  |  |
| Attenuated psychotic negative symptoms | n. a. |  |  |  |  |  |
| NOS | 7 | -0.0169 | 0.0623 | -0.2711 | 0.7863 | -0.1389, 0.1052 |
| Baseline AP exposure | n. a. |  |  |  |  |  |
| Type of CHR-P instrument | n.a |  |  |  |  |  |

**References**

1. Cochrane Handbook for Systematic Reviews of Interventions. Version 5.1.0. [updated March 2011]: The Cochrane Collaboration; 2011 [Available from: Available from <www.handbook.cochrane.org>.

2. Borenstein M, Hedges LV, Higgins JPT, Rothstein HR. Part 5. Chapter 22. Complex Data Structures Introduction to Meta-Analysis: John Wiley & Sons, Ltd. ; 2009.

3. Egger M, Davey Smith G, Schneider M, Minder C. Bias in meta-analysis detected by a simple, graphical test. BMJ (Clinical research ed). 1997;315(7109):¡629-34.

4. Fusar-Poli P, Deste G, Smieskova R, Barlati S, Yung AR, Howes O, et al. Cognitive Functioning in Prodromal Psychosis A Meta-analysis. Archives of General Psychiatry. 2012;69(6):562-71.

5. Hauser M, Zhang J-P, Sheridan EM, Burdick KE, Mogil R, Kane JM, et al. Neuropsychological Test Performance to Enhance Identification of Subjects at Clinical High Risk for Psychosis and to Be Most Promising for Predictive Algorithms for Conversion to Psychosis: A Meta-Analysis. Journal of Clinical Psychiatry. 2017;78(1):E28-E40.

6. Reitan RM, Wolfson D. The Halstead–Reitan Neuropsycholgical Test Battery: Therapy and clinical interpretation. Tucson, AZ: Neuropsychological Press.; 1985.

7. Keefe RSE. Brief Assessment of Cognition in Schizophrenia (BACS) Manual—A: Version 2.1. Durham, NC: Duke University Medical Center; 1999.

8. Spreen O, Strauss E. A Compendium of Neuropsychological Tests. New York: xford University; 1991.

9. Thurstone LL. Primary mental abilities. Psychometric Monographs. 1938;1.

10. Wechsler D. Wechsler Adult Intelligence Scale—3rd ed (WAISIII): Administration and Scoring Manual. San Antonio, Tex: Psychological Corp; 1997.

11. Golden CJ. Stroop Color and Word Test. Chicago, Illinois: Stoelting Company; 1978.

12. Nuechterlei KH, Edell WS, Norris M, Dawson ME. Attentionalvulnerability indicators, thought disorder, and negative symptoms. Schizophrenia bulletin. 1986;12:408–26.

13. Cornblatt BA, Risch NJ, Faris G, Friedman D, Erlenmeyer-Kimling L. The Continuous Performance Test, Identical Pairs version (CPT-IP), I: new findings about sustained attention in normal families. Psychiatry research. 1988;26:223-38.

14. Wechsler D. The Wechsler Memory Scale, 3rd ed. . San Antonio, Tex: Psychological Corp (Harcourt); 1997.

15. Gold JM, Carpenter C, Randolph C, Goldberg TE, Weinberger DR. Auditory working memory and Wisconsin Card Sorting Test performance in schizophrenia. Archives of general psychiatry. 1997;54:159–65.

16. Wechsler D. Wechsler Abbreviated Scale of Intelligence. San Antonio, TX.: Psychological Corporation; 1999.

17. Petrides M, Alivisatos B, Evans AC. Functional activation of the human ventrolateral frontal cortex during mnemonic retrieval of verbal information. Proc Natl Acad Sci U S A. 1995;92(13):5803-7.

18. Brandt J, R.H.B. B. The Hopkins Verbal Learning Test—Revised: Professional Manual. . Odessa, Fla: Psychological AssessmentcResources, Inc.; 2001.

19. Lezak MD. Neuropsychological Assessment: Oxford University Press; 2004.

20. Woods SP, Delis DC, Scott JC, Kramer JH, Holdnack JA. The California Verbal Learning Test-second edition: Test-retest reliability, practice effects, and reliable change indices for the standard and alternate forms. Archives of clinical neuropsychology : the official journal of the National Academy of Neuropsychologists. 2006;21:413-20.

21. Delis DC, Kramer JH, Kaplan E, Ober BA. California Verbal Learning Test Manual — II: The Psychological Corporation, San Antonio, TX; 2000.

22. Benedict RHB. Benedict RHB: Brief Visuospatial Memory Test—Revised: Professional Manual. Odessa, Fla: Psychological Assessment Resources, Inc.; 1997.

23. Rey A. L’examen clinique en psychologie. Presses Un, Parisi, M.1964.

24. White T, Stern RA. Neuropsychological Assessment Battery: Psychometric and Technical Manual. Lutz, Fla: Psychological Assessment Resources, Inc; 2003.

25. Baron-Cohen S, Jolliffe T, Mortimore C, Robertson M. Another advanced test of theory of mind: evidence from very high functioning adults with autism or asperger syndrome. J Child Psychol Psychiatry. 1997;38(7):813-22.

26. Baron-Cohen S, Wheelwright S, Hill J, Raste Y, Plumb I. The "Reading the Mind in the Eyes" Test revised version: a study with normal adults, and adults with Asperger syndrome or high-functioning autism. J Child Psychol Psychiatry. 2001;42(2):241-51.

27. van 't Wout M, Aleman A, Kessels RP, Laroi F, Kahn RS. Emotional processing in a non-clinical psychosis-prone sample. Schizophrenia research. 2004;68(2-3):271-81.

28. Corcoran R, Mercer G, Frith CD. Schizophrenia, symptomatology and social inference: Investigating “theory of mind” in people with schizophrenia. Schizophrenia research. 1995;17:5-13.

29. Wechsler D. WAIS-R : Wechsler adult intelligence scale-revised. New York, N.Y. : Psychological Corporation; 1981.

30. Wechsler D. Wechsler Intelligence Scale for Children, 3rd edition New York, N. Y,: Psychological Corporation; 1991.

31. Nelson HE. National Adult Reading Test (NART): Test manual. Windsor: NFER-Nelson.; 1982.

32. Maurer K, Hafner H. [Early diagnosis of schizophrenia]. MMW Fortschritte der Medizin. 2007;149(13):36-8.

33. Grant DA. Computer version of the Wisconsin Card Sorting Test, WCST. Odessa, Fla: Psychological Assessment Resources; 2000.

34. Stroop JR. Studies of interference in serial verbal reactions. J Exp Psychol. 1935;18:643-62.

35. Rombouts RP. Finger Tapping Test. In: software] CC, editor.: Lelystad: BuroTesteR; 2002.

36. Doty RL, Shaman P, Kimmelman CP, Dann MS. University of Pennsylvania Smell Identification Test: a rapid quantitative olfactory function test for the clinic. Laryngoscope. 1984;94(2 Pt 1):176-8.

37. Addington J, Piskulic D, Perkins D, Woods SW, Liu L, Penn DL. Affect recognition in people at clinical high risk of psychosis. Schizophrenia research. 2012;140(1-3):87-92.

38. Addington J, Liu L, Perkins DO, Carrion RE, Keefe RS, Woods SW. The Role of Cognition and Social Functioning as Predictors in the Transition to Psychosis for Youth With Attenuated Psychotic Symptoms. Schizophrenia bulletin. 2017;43(1):57-63.

39. Atkinson RJ, Fulham WR, Michie PT, Ward PB, Todd J, Stain H, et al. Electrophysiological, cognitive and clinical profiles of at-risk mental state: The longitudinal Minds in Transition (MinT) study. PloS one. 2017;12(2).

40. Becker HE, Nieman DH, Wiltink S, Dingemans PM, de Fliert JRv, Velthorst E, et al. Neurocognitive functioning before and after the first psychotic episode: does psychosis result in cognitive deterioration? Psychological medicine. 2010;40(10):1599-606.

41. Blanchard MM, Jacobson S, Clarke MC, Connor D, Kelleher I, Garavan H, et al. Language, motor and speed of processing deficits in adolescents with subclinical psychotic symptoms. Schizophrenia research. 2010;123(1):71-6.

42. Bolt LK, Amminger GP, Farhall J, McGorry PD, Nelson B, Markulev C, et al. Neurocognition as a predictor of transition to psychotic disorder and functional outcomes in ultra-high risk participants: Findings from the NEURAPRO randomized clinical trial. Schizophrenia research. 2019;206:67-74.

43. Brewer WJ, Wood SJ, McGorry PD, Francey SM, Phillips LJ, Yung AR, et al. Impairment of olfactory identification ability in individuals at ultra-high risk for psychosis who later develop schizophrenia. The American journal of psychiatry. 2003;160(10):1790-4.

44. Brockhaus-Dumke A, Tendolkar I, Pukrop R, Schultze-Lutter F, Klosterkötter J, Ruhrmann S. Impaired mismatch negativity generation in prodromal subjects and patients with schizophrenia. Schizophrenia research. 2005;73(2-3):297-310.

45. Broome M, Day F, Valli I, Valmaggiaa L, Johns L, Howes O, et al. Delusional ideation, manic symptomatology and working memory in a cohort at clinical high-risk for psychosis: a longitudinal study. . European psychiatry : the journal of the Association of European Psychiatrists. 2012;27:258–63.

46. Chu AOK, Chang WC, Chan SKW, Lee EHM, Hui CLM, Chen EYH. Comparison of cognitive functions between first-episode schizophrenia patients, their unaffected siblings and individuals at clinical high-risk for psychosis. Psychological medicine. 2019;49(11):1929-36.

47. Chung YS, Kang D-H, Shin NY, Yoo SY, Kwon JS. Deficit of theory of mind in individuals at ultra-high-risk for schizophrenia. Schizophrenia research. 2008;99(1-3):111-8.

48. Corcoran CM, Keilp JG, Kayser J, Klim C, Butler PD, Bruder GE, et al. Emotion recognition deficits as predictors of transition in individuals at clinical high risk for schizophrenia: a neurodevelopmental perspective. Psychological medicine. 2015;45(14):2959-73.

49. Couture SM, Penn DL, Addington J, Woods SW, Perkins DO. Assessment of social judgments and complex mental states in the early phases of psychosis. Schizophrenia research. 2008;100(1-3):237-41.

50. Cui H, Giuliano AJ, Zhang T, Xu L, Wei Y, Tang Y, et al. Cognitive dysfunction in a psychotropic medication-naive, clinical high-risk sample from the ShangHai-At-Risk-for-Psychosis (SHARP) study: Associations with clinical outcomes. Schizophrenia research. 2020.

51. Eastvold AD, Heaton RK, Cadenhead KS. Neurocognitive deficits in the (putative) prodrome and first episode of psychosis. Schizophrenia research. 2007;93(1-3):266-77.

52. Egloff L, Lenz C, Studerus E, Heitz U, Harrisberger F, Smieskova R, et al. No associations between medial temporal lobe volumes and verbal learning/memory in emerging psychosis. European Journal of Neuroscience. 2019;50(6):3060-71.

53. Eisenacher S, Rausch F, Ainser F, Englisch S, Becker A, Mier D, et al. Early cognitive basic symptoms are accompanied by neurocognitive impairment in patients with an 'at-risk mental state' for psychosis. Early intervention in psychiatry. 2018;12(4):586-95.

54. Epstein KA, Cullen KR, Mueller BA, Robinson P, Lee S, Kumra S. White matter abnormalities and cognitive impairment in early-onset schizophrenia-spectrum disorders. Journal of the American Academy of Child and Adolescent Psychiatry. 2014;53(3):362-72.e1-2.

55. Frommann I, Pukrop R, Brinkmeyer J, Bechdolf A, Ruhrmann S, Berning J, et al. Neuropsychological Profiles in Different At-Risk States of Psychosis: Executive Control Impairment in the Early-and Additional Memory Dysfunction in the Late-Prodromal State. Schizophrenia bulletin. 2011;37(4):861-73.

56. Gill KE, Evans E, Kayser J, Ben-David S, Messinger J, Bruder G, et al. Smell identification in individuals at clinical high risk for schizophrenia. Psychiatry Research 2014;220(1-2):201-4.

57. Goghari VM, Brett C, Tabraham P, Johns L, Valmaggia L, Broome M, et al. Spatial working memory ability in individuals at ultra high risk for psychosis. Journal of psychiatric research. 2014;50:100-5.

58. Gupta T, Mittal VA. Nicotine usage is associated with elevated processing speed, spatial working memory, and visual learning performance in youth at ultrahigh-risk for psychosis. Psychiatry research. 2014;220(1-2):687-90.

59. He Y, Li Z, Ma X, Yuan L, Ouyang L, Tang J, et al. Olfactory and cognitive functions in Chinese individuals at clinical high risk for psychosis. Psychiatry research. 2019;272:51-3.

60. Healey KM, Penn DL, Perkins D, Woods SW, Addington J. Theory of mind and social judgments in people at clinical high risk of psychosis. Schizophrenia research. 2013;150(2-3):498-504.

61. Hou CL, Xiang YT, Wang ZL, Everall I, Tang Y, Yang C, et al. Cognitive functioning in individuals at ultra-high risk for psychosis, first-degree relatives of patients with psychosis and patients with first-episode schizophrenia. Schizophrenia research. 2016;174(1-3):71-6.

62. Hur JW, Shin NY, Jang JH, Shim G, Park HY, Hwang JY, et al. Clinical and neurocognitive profiles of subjects at high risk for psychosis with and without obsessive-compulsive symptoms. The Australian and New Zealand journal of psychiatry. 2012;46(2):161-9.

63. Hur JW, Byun MS, Shin NY, Shin YS, Kim SN, Jang JH, et al. General intellectual functioning as a buffer against theory-of-mind deficits in individuals at ultra-high risk for psychosis. Schizophrenia research. 2013;149(1-3):83-7.

64. Hwang WJ, Lee TY, Shin WG, Kim M, Kim J, Lee J, et al. Global and Specific Profiles of Executive Functioning in Prodromal and Early Psychosis. Frontiers in psychiatry. 2019;10:356.

65. Ilonen T, Heinimaa M, Korkeila J, Svirskis T, Salokangas RKR. Differentiating adolescents at clinical high risk for psychosis from psychotic and non-psychotic patients with the Rorschach. Psychiatry research. 2010;179(2):151-6.

66. Jahshan C, Heaton RK, Golshan S, Cadenhead KS. Course of Neurocognitive Deficits in the Prodrorne and First Episode of Schizophrenia. Neuropsychology. 2010;24(1):109-20.

67. Kamath V, Turetsky BI, Calkins ME, Kohler CG, Conroy CG, Borgmann-Winter K, et al. Olfactory processing in schizophrenia, non-ill first-degree family members, and young people at-risk for psychosis. The world journal of biological psychiatry : the official journal of the World Federation of Societies of Biological Psychiatry. 2014;15(3):209-18.

68. Kang M, Bang M, Lee SY, Lee E, Yoo SW, An SK. Coping styles in individuals at ultra-high risk for psychosis: Associations with cognitive appraisals. Psychiatry research. 2018;264:162-8.

69. Kim HS, Shin NY, Jang JH, Kim E, Shim G, Park HY, et al. Social cognition and neurocognition as predictors of conversion to psychosis in individuals at ultra-high risk. Schizophrenia research. 2011;130(1-3):170-5.

70. Kim D, Lee S, Choi M, Youn H, Suh S, Jeong HG, et al. Diffusion tensor imaging reveals abnormal brain networks in elderly subjects with subjective cognitive deficits. Neurological sciences : official journal of the Italian Neurological Society and of the Italian Society of Clinical Neurophysiology. 2019;40(11):2333-42.

71. Koren D, Scheyer R, Stern Y, Adres M, Reznik N, Apter A, et al. Metacognition strengthens the association between neurocognition and attenuated psychosis syndrome: Preliminary evidence from a pilot study among treatment-seeking versus healthy adolescents. Schizophrenia research. 2019;210:207-14.

72. Korver N, Nieman DH, Becker HE, van de Fliert JR, Dingemans PH, de Haan L, et al. Symptomatology and neuropsychological functioning in cannabis using subjects at ultra-high risk for developing psychosis and healthy controls. The Australian and New Zealand journal of psychiatry. 2010;44(3):230-6.

73. Koshiyama D, Kirihara K, Tada M, Nagai T, Fujioka M, Koike S, et al. Association between mismatch negativity and global functioning is specific to duration deviance in early stages of psychosis. Schizophrenia research. 2018;195:378-84.

74. Koutsouleris N, Davatzikos C, Bottlender R, Patschurek-Kliche K, Scheuerecker J, Decker P, et al. Early recognition and disease prediction in the at-risk mental states for psychosis using neurocognitive pattern classification. Schizophrenia bulletin. 2012;38(6):1200-15.

75. Kristensen TD, Mandl RCW, Raghaya JM, Jessen K, Jepsen JRM, Fagerlunc B, et al. Widespread higher fractional anisotropy associates to better cognitive functions in individuals at ultra-high risk for psychosis. Human brain mapping. 2019;40(18):5185-201.

76. Lee TY, Shin YS, Shin NY, Kim SN, Jang JH, Kang DH, et al. Neurocognitive function as a possible marker for remission from clinical high risk for psychosis. Schizophrenia research. 2014;153(1-3):48-53.

77. Lee SY, Bang M, Kim KR, Lee MK, Park JY, Song YY, et al. Impaired facial emotion recognition in individuals at ultra-high risk for psychosis and with first-episode schizophrenia, and their associations with neurocognitive deficits and self-reported schizotypy. Schizophrenia research. 2015;165(1):60-5.

78. Lepock JR, Ahmed S, Mizrahi R, Gerritsen CJ, Maheandiran M, Drvaric L, et al. Relationships between cognitive event-related brain potential measures in patients at clinical high risk for psychosis. Schizophrenia research. 2019.

79. Li RR, Lyu HL, Liu F, Lian N, Wu RR, Zhao JP, et al. Altered functional connectivity strength and its correlations with cognitive function in subjects with ultra-high risk for psychosis at rest. CNS neuroscience & therapeutics. 2018;24(12):1140-8.

80. Lin A, Yung AR, Nelson B, Brewer WJ, Riley R, Simmons M, et al. Neurocognitive predictors of transition to psychosis: medium- to long-term findings from a sample at ultra-high risk for psychosis. Psychological medicine. 2013;43(11):2349-60.

81. Lindgren M, Manninen M, Laajasalo T, Mustonen U, Kalska H, Suvisaari J, et al. The relationship between psychotic-like symptoms and neurocognitive performance in a general adolescent psychiatric sample. Schizophrenia research. 2010;123(1):77-85.

82. Liu Y, Wang G, Jin H, Lyu H, Liu Y, Guo W, et al. Cognitive deficits in subjects at risk for psychosis, first-episode and chronic schizophrenia patients. Psychiatry research. 2019;274:235-42.

83. Magaud E, Morvan Y, Rampazzo A, Alexandre C, Willard D, Gaillard R, et al. Subjects at Ultra High Risk for psychosis have 'heterogeneous' intellectual functioning profile: a multiple-case study. Schizophrenia research. 2014;152(2-3):415-20.

84. Menghini-Muller S, Studerus E, Ittig S, Valmaggia LR, Kempton MJ, van der Gaag M, et al. Sex differences in cognitive functioning of patients at-risk for psychosis and healthy controls: Results from the European Gene-Environment Interactions study. European psychiatry : the journal of the Association of European Psychiatrists. 2020;63(1):e25-e.

85. Metzler S, Dvorsky D, Wyss C, Müller M, Gerstenberg M, Traber-Walker N, et al. Changes in neurocognitive functioning during transition to manifest disease: comparison of individuals at risk for schizophrenic and bipolar affective psychoses. Psychological medicine. 2015;45(10):2123-34.

86. Millman ZB, Goss J, Schiffman J, Mejias J, Gupta T, Mittal VA. Mismatch and lexical retrieval gestures are associated with visual information processing, verbal production, and symptomatology in youth at high risk for psychosis. Schizophrenia research. 2014;158(1-3):64-8.

87. Mirzakhanian H, Singh F, Seeber K, Shafer KM, Cadenhead KS. A Developmental Look at the Attentional System in the At Risk and First Episode of Psychosis: Age related Changes in Attention along the Psychosis Spectrum. Neurocognitive Neuropsychiatry. 2013;18(0):26–43.

88. Mittal VA, Walker EF, Bearden CE, Walder D, Trottman H, Daley M, et al. Markers of basal ganglia dysfunction and conversion to psychosis: neurocognitive deficits and dyskinesias in the prodromal period. Biological psychiatry. 2010;68(1):93-9.

89. Modinos G, McGuire P. The prodromal phase of psychosis. Current Opinion in Neurobiology. 2015;30:100-5.

90. Montalvo I, Gutierrez-Zotes A, Creus M, Monseny R, Ortega L, Franch J, et al. Increased prolactin levels are associated with impaired processing speed in subjects with early psychosis. PloS one. 2014;9(2):e89428.

91. Ohmuro N, Katsura M, Obara C, Kikuchi T, Sakuma A, Iizuka K, et al. Deficits of cognitive theory of mind and its relationship with functioning in individuals with an at-risk mental state and first-episode psychosis. Psychiatry research. 2016;243:318-25.

92. Ohmuro N, Katsura M, Obara C, Kikuchi T, Hamaie Y, Sakuma A, et al. The relationship between cognitive insight and cognitive performance among individuals with at-risk mental state for developing psychosis. Schizophrenia research. 2018;192:281-6.

93. Pflueger MO, Calabrese P, Studerus E, Zimmermann R, Gschwandtner U, Borgwardt S, et al. The neuropsychology of emerging psychosis and the role of working memory in episodic memory encoding. Psychology research and behavior management. 2018;11:157-68.

94. Pukrop R, Schultze-Lutter F, Ruhrmann S, Brockhaus-Dumke A, Tendolkar I, Bechdolf A, et al. Neurocognitive functioning in subjects at risk for a first episode of psychosis compared with first- and multiple-episode schizophrenia. Journal of clinical and experimental neuropsychology. 2006;28(8):1388-407.

95. Pukrop R, Ruhrmann S, Schultze-Lutter F, Bechdolf A, Brockhaus-Dumke A, Klosterkötter J. Neurocognitive indicators for a conversion to psychosis: comparison of patients in a potentially initial prodromal state who did or did not convert to a psychosis. Schizophrenia research. 2007;92(1-3):116-25.

96. Randers L, Jepsen JRM, Fagerlund B, Nordholm D, Krakauer K, Hjorthoj C, et al. Associations between facial affect recognition and neurocognition in subjects at ultra-high risk for psychosis: A case-control study. Psychiatry research. 2020;290:112969.

97. Sanada K, Ruiz de Azua S, Nakajima S, Alberich S, Ugarte A, Zugasti J, et al. Correlates of neurocognitive functions in individuals at ultra-high risk for psychosis - A 6-month follow-up study. Psychiatry research. 2018;268:1-7.

98. Seidman LJ, Giuliano AJ, Meyer EC, Addington J, Cadenhead KS, Cannon TD, et al. Neuropsychology of the Prodrome to Psychosis in the NAPLS Consortium Relationship to Family History and Conversion to Psychosis. Archives of general psychiatry. 2010;67(6):578-88.

99. Seidman LJ, Shapiro DI, Stone WS, Woodberry KA, Ronzio A, Cornblatt BA, et al. Association of Neurocognition With Transition to Psychosis: Baseline Functioning in the Second Phase of the North American Prodrome Longitudinal Study. JAMA psychiatry. 2016;73(12):1239-48.

100. Shin YS, Kim SY, Lee TY, Hur JW, Shin NY, Kim SN, et al. Longitudinal change in neurocognition and its relation to symptomatic and functional changes over 2years in individuals at clinical high-risk for psychosis. Schizophrenia research. 2016;174(1-3):50-7.

101. Simon AE, Cattapan-Ludewig K, Zmilacher S, Arbach D, Gruber K, Dvorsky DN, et al. Cognitive functioning in the schizophrenia prodrome. Schizophrenia bulletin. 2007;33(3):761-71.

102. Standford AD, Messinger J, Malaspina D, Corcoran C. Theory of Mind in patients at clinical high risk for psychosis. Schizophrenia research. 2011;131:11–7.

103. Studerus E, Corbisiero S, Mazzariello N, Ittig S, Leanza L, Egloff L, et al. Can neuropsychological testing facilitate differential diagnosis between at-risk mental state (ARMS) for psychosis and adult attention-deficit/hyperactivity disorder (ADHD)? European psychiatry : the journal of the Association of European Psychiatrists. 2018;52:38-44.

104. Szily E, Kéri S. Anomalous subjective experience and psychosis risk in young depressed patients. Psychopathology. 2009;42(4):229-35.

105. Takahashi T, Nakamura M, Sasabayashi D, Komori Y, Higuchi Y, Nishikawa Y, et al. Olfactory deficits in individuals at risk for psychosis and patients with schizophrenia: relationship with socio-cognitive functions and symptom severity. European archives of psychiatry and clinical neuroscience. 2018;268(7):689-98.

106. Thompson A, Papas A, Bartholomeusz C, Allott K, Amminger GP, Nelson B, et al. Social cognition in clinical "at risk" for psychosis and first episode psychosis populations. Schizophrenia research. 2012;141(2-3):204-9.

107. Tognin S, Catalan A, Modinos G, Kempton MJ, Bilbao A, Nelson B, et al. Emotion Recognition and Adverse Childhood Experiences in Individuals at Clinical High Risk of Psychosis. Schizophrenia bulletin. 2020;46(4):823-33.

108. Tor J, Dolz M, Sintes-Estevez A, de la Serna E, Puig O, Munoz-Samons D, et al. Neuropsychological profile of children and adolescents with psychosis risk syndrome: the CAPRIS study. European child & adolescent psychiatry. 2020.

109. Üçok A, Direk N, Koyuncu A, Keskin-Ergen Y, Yüksel Ç, Güler J, et al. Cognitive deficits in clinical and familial high risk groups for psychosis are common as in first episode schizophrenia. Schizophrenia research. 2013;151(1-3):265-9.

110. van Rijn S, Aleman A, de Sonneville L, Sprong M, Ziermans T, Schothorst P, et al. Misattribution of facial expressions of emotion in adolescents at increased risk of psychosis: the role of inhibitory control. Psychological medicine. 2011;41(3):499-508.

111. Wood SJ, Brewer WJ, Koutsouradis P, Phillips LJ, Francey SM, Proffitt TM, et al. Cognitive decline following psychosis onset - Data from the PACE clinic. British Journal of Psychiatry. 2007;191:S52-S7.

112. Woodberry KA, McFarlane WR, Giuliano AJ, Verdi MB, Cook WL, Faraone SV, et al. Change in neuropsychological functioning over one year in youth at clinical high risk for psychosis. Schizophrenia research. 2013;146(1-3):87-94.

113. Zhang T, Cui H, Tang Y, Xu L, Li H, Wei Y, et al. Correlation of social cognition and neurocognition on psychotic outcome: a naturalistic follow-up study of subjects with attenuated psychosis syndrome. Scientific reports. 2016;6.

114. Ziermans T, de Wit S, Schothorst P, Sprong M, van Engeland H, Kahn R, et al. Neurocognitive and clinical predictors of long-term outcome in adolescents at ultra-high risk for psychosis: a 6-year follow-up. PloS one. 2014;9(4):e93994.
